# Supplementary material for: The impact of ancestral, genetic, and environmental influences on germline de novo mutation rates and spectra
Source: Nat Commun. 2025 May 15;16:4527. doi: 10.1038/s41467-025-59750-x (PMC12081642; doi:10.1038/s41467-025-59750-x)
Supplement: Supplementary file 1 — Supplementary Information [file 41467_2025_59750_MOESM1_ESM.pdf]

# Supplementary information

**Supplementary Figures** .......... **2**
**Supplementary Notes** .......... **24**
Supplementary Note 1. Ensuring that the associations between ancestry and DNM counts are not due to technical artefacts or ascertainment bias .......... 24
A) Ancestral biases in the reference genome .......... 24
B) Biases due to ancestry-differential rates of post-zygotic mutations .......... 24
C) Biases due to ancestrally-differential rates of missed constitutive heterozygous calls in parents .......... 25
D) Ancestry-related ascertainment biases into the dataset .......... 26
E) Biases due to differences in parental age between ancestry groups .......... 27
Supplementary Note 2. Comparing ancestry-associated differences on mutation spectra using DNM data and polymorphism data .......... 29
Supplementary Note 3. Checking ancestry specific DNM spatial distribution in the human genome .......... 31
Supplementary Note 4. Attempting to identify associations between parental smoking behaviour and DNM mutation spectra .......... 32
Supplementary Note 5. Estimating power to detect spectra differences in GEL DNMs as compared to polymorphism data .......... 34
Supplementary Note 6. Cross-parental effects on early embryonic mutations .......... 35
Supplementary Note 7. Modelling mean-variance overdispersion for DNM count data .......... 36
**Supplementary References** .......... **37**

## Supplementary Figures

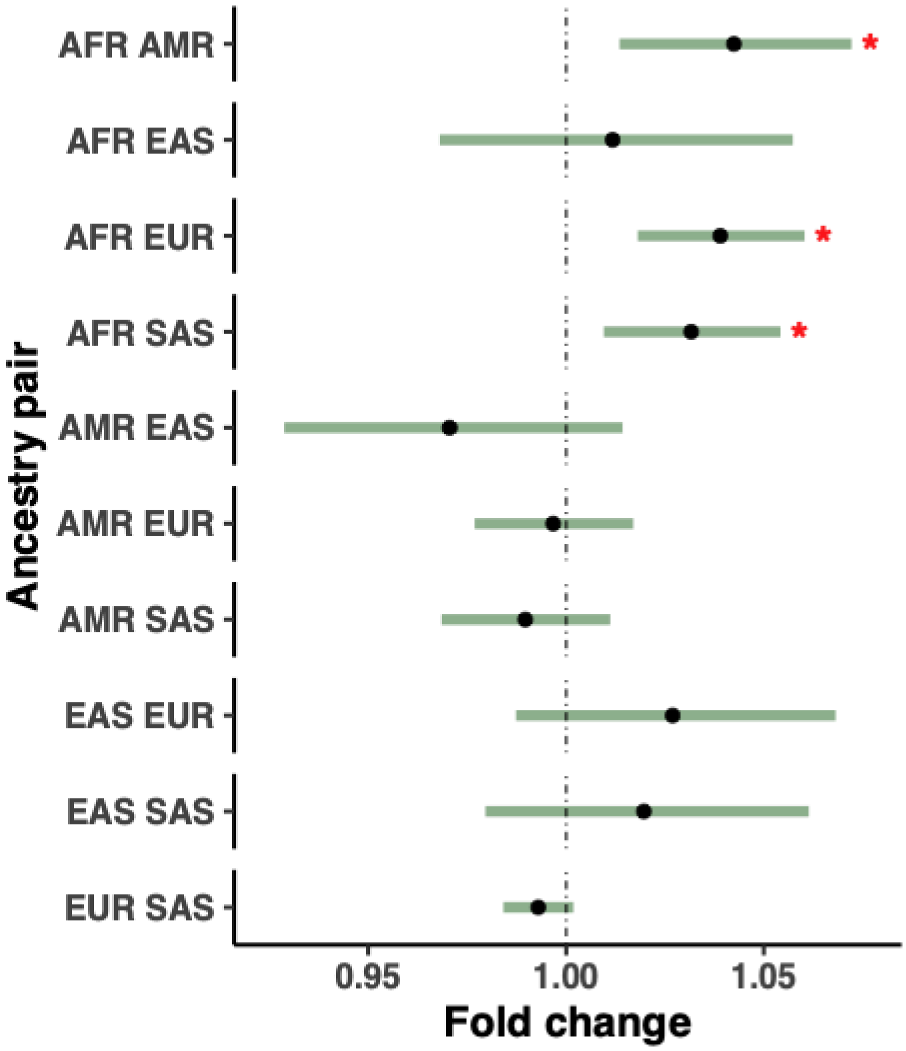


**Supplementary Figure 1. Pairwise comparison of ancestry associated differences on DNM counts per trio.** Showing fold change difference estimates on DNM counts between ancestry pairs (generalised linear regression, **Model 1**, Methods). Effect estimate corresponds to the first ancestry in each row pair as compared to the second. Bars correspond to two-tailed 95% confidence intervals for the effect estimate. Asterisks indicate significant associations after multi-testing correction (5% FDR) for all non-redundant pairwise tests (n tests = 10).

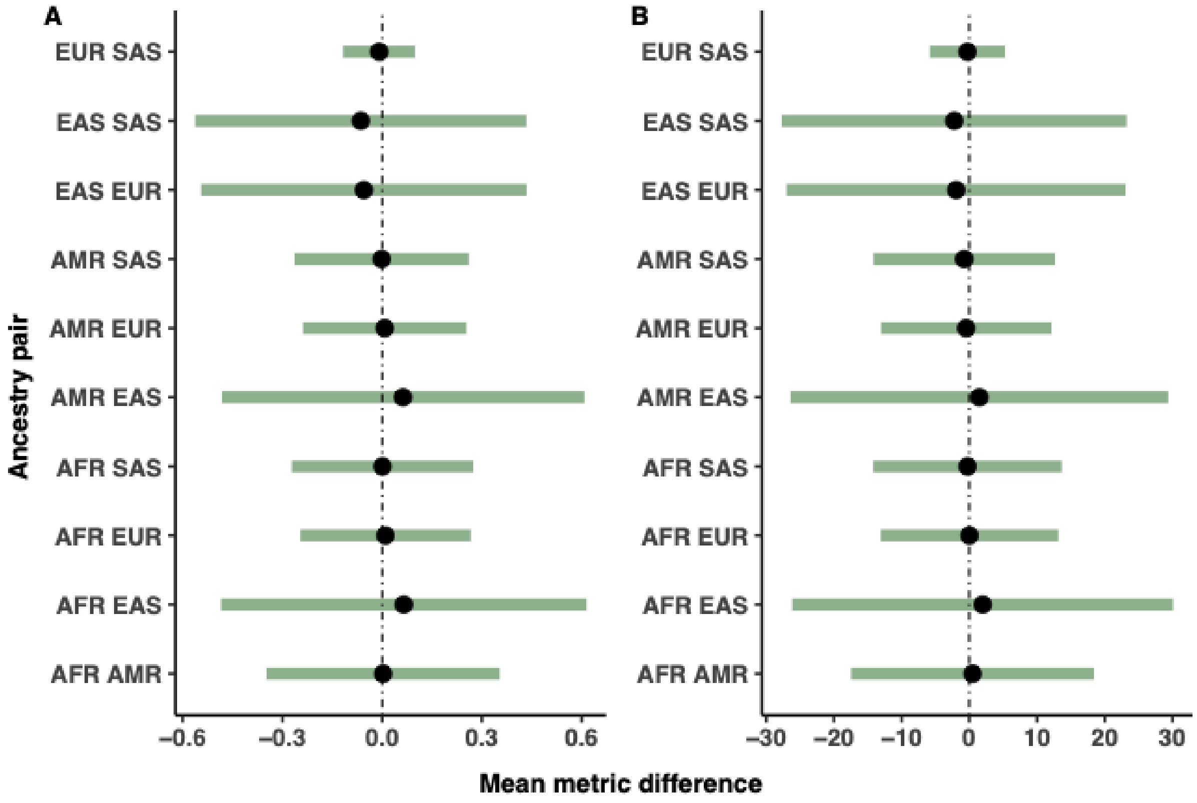


**Supplementary Figure 2. Pairwise comparisons of average NM (avgNM) metrics across ancestries.** Comparing **A)** Per trio average avgNM per DNM. **B)** Per trio maximum avgNM per DNM. Bars correspond to two-tailed 95% confidence intervals for the mean difference. Effect direction corresponds to the first ancestry in each row pair. No significant differences were found for any ancestry pair or metric (ANOVA followed by Tukey HSD test; two-sided $p > 0.05$).

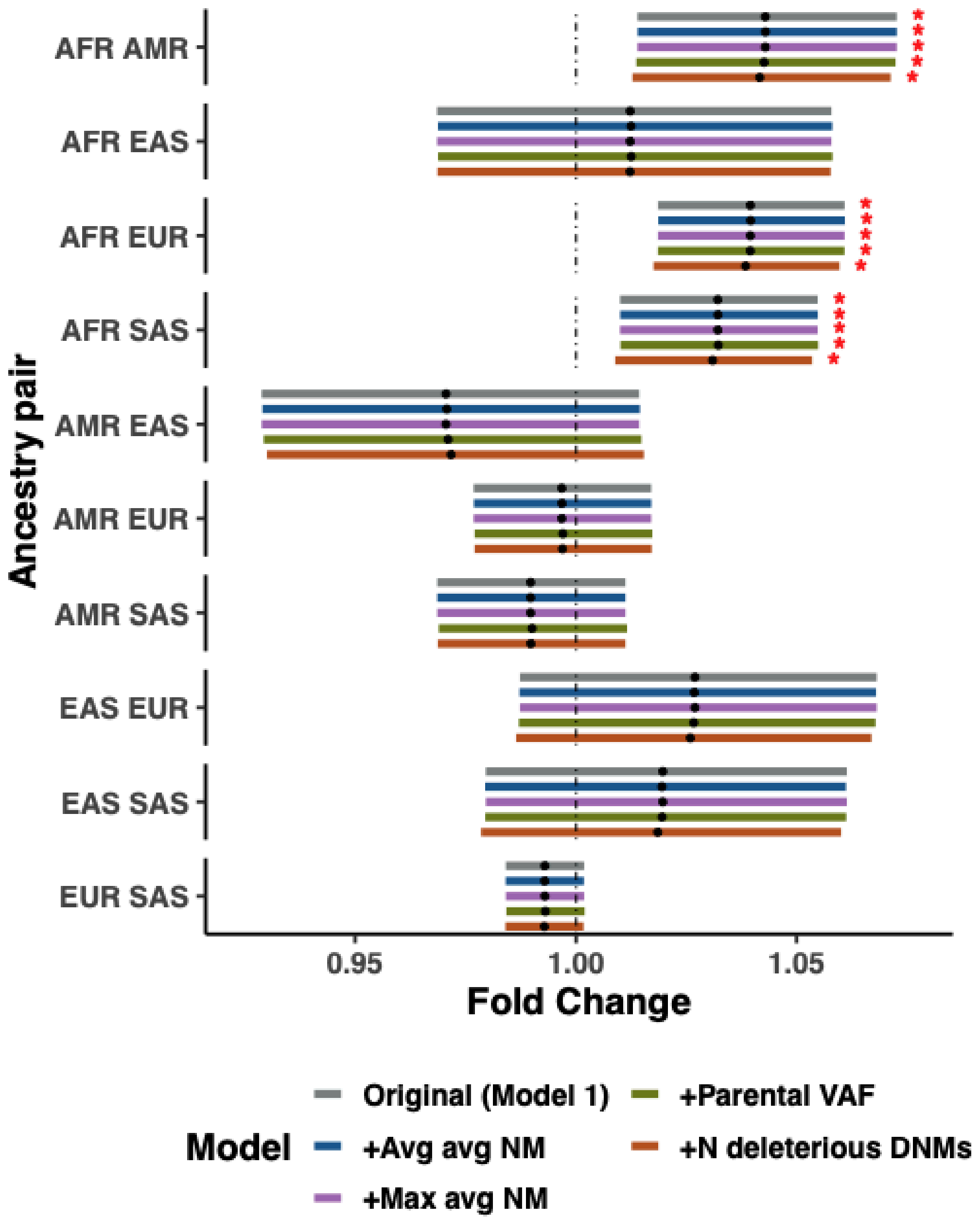


**Supplementary Figure 3. Pairwise comparison of ancestry associated differences on DNM counts per trio before and after controlling for covariates capturing potential artifacts.** Bar colours represent an independent run of **Model 1** including an extra covariate capturing a potential artifactual source. The extra covariates tested were: average average mismatches per read per trio (avg avg NM), maximum average mismatches per read per trio (max avg NM), mean parental variant allele fraction per trio (parental VAF), or number of potentially deleterious *de novo* variants (n deleterious DNMs). Showing fold change differences in DNM counts between ancestry pairs. The grey bar corresponds to the effect estimate from the original **Model 1**. Effect estimate corresponds to the first ancestry in each row pair relative to the second. Bars correspond to two-tailed 95% confidence intervals for the effect estimate. Asterisks indicate significant fold change differences in DNM counts for the ancestry pair after multi-testing correction in a single experiment (n tests per experiment = 10, 5% FDR).

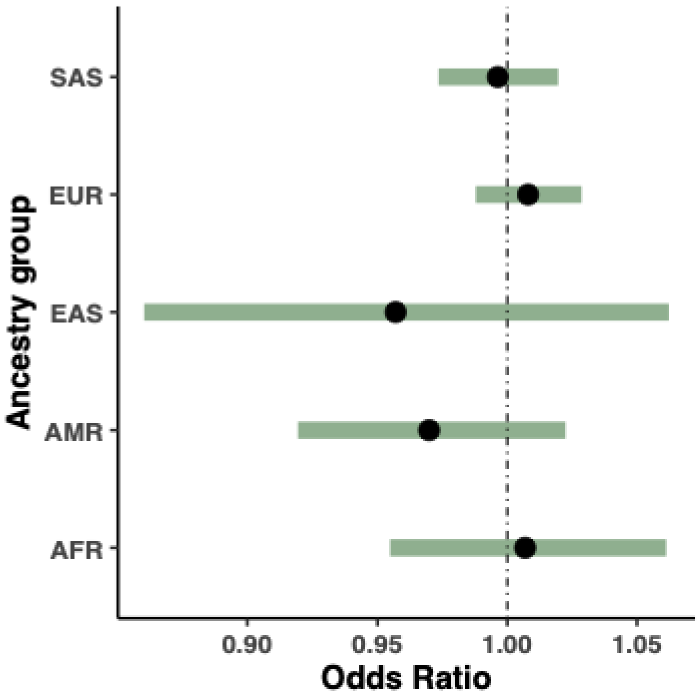


**Supplementary Figure 4.Comparison of relative abundance of putative post-zygotic mutation events across ancestry groups.** Showing the odds ratio estimate for the presence of putative postzygotic mutation events (PZMs) in trios from a given ancestry as compared to the rest. Bars represent two-tailed 95% confidence intervals. No significant enrichment (or depletion) of PZM events was found for any ancestry category (Fisher exact test; two-sided $p > 0.05$).

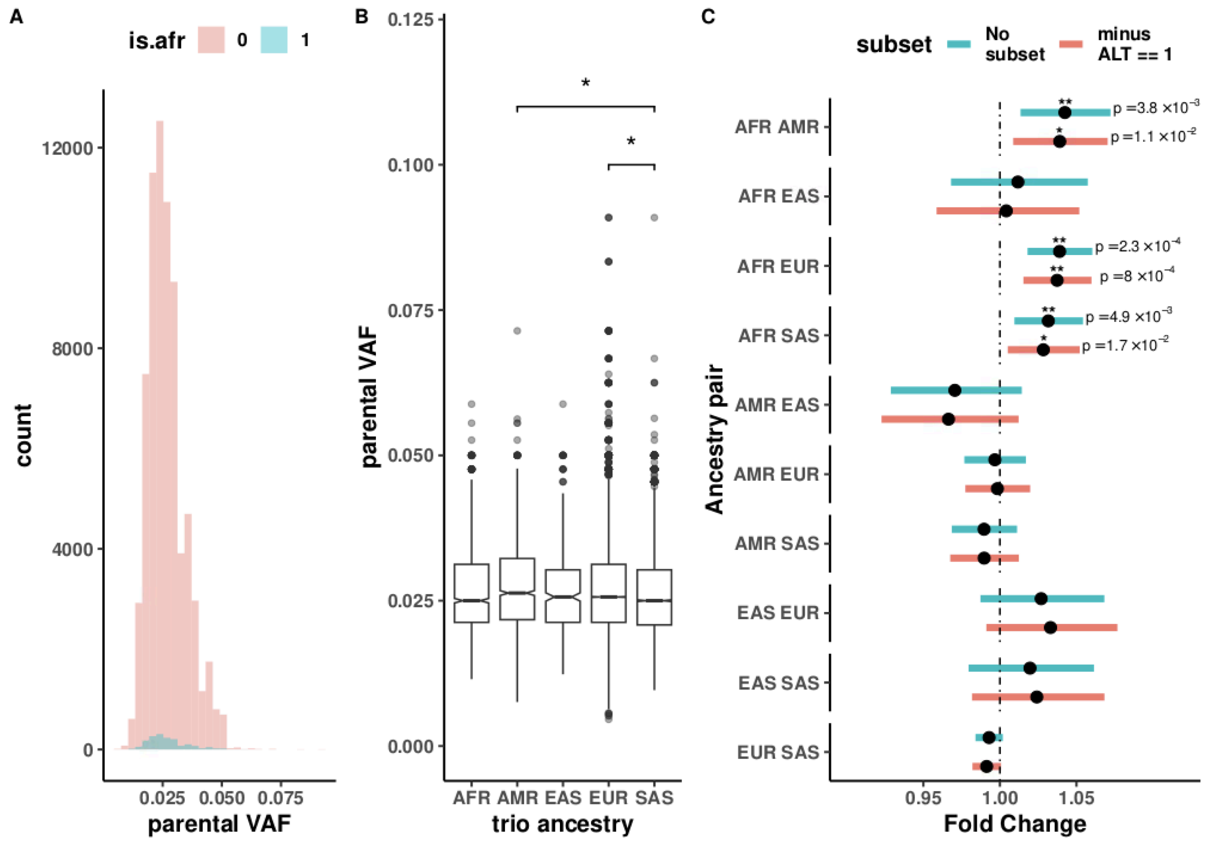


**Supplementary Figure 5. Exploring whether missed heterozygous calls in parents could be driving the ancestry differences in DNM rate. A)** Distribution of parental VAFs for putative HET sites in AFR trios (is.afr == 1; n putative HETs = 1,612) versus those in any other ancestry (is.afr == 0; n putative HETs = 71,488). No significant differences of VAFs were detected (Wilcoxon rank-sum test $p > 0.05$). **B)** Pairwise comparison of parental VAFs for putative HET sites stratified by ancestry. Lines and asterisks indicate significant differences for the indicated ancestry pair (ANOVA followed by Tukey HSD test $p \leq 0.05$). **C)** Pairwise comparison of ancestry-associated differences in DNM counts using the whole DNM dataset (n DNMs = 639,361) or the subset excluding DNMs with parental ALT allele counts > 0 (n = 566,258). Bars correspond to two-tailed 95% confidence intervals for the effect estimate. Double asterisks indicate significant fold change differences in DNM counts for the ancestry pair after multi-testing correction in a single experiment (n tests per experiment = 10, 5% FDR). A single asterisk represents nominally significant differences (unadjusted $p \leq 0.05$). Nominal p values are indicated in text for all comparisons reaching at least nominal significance.

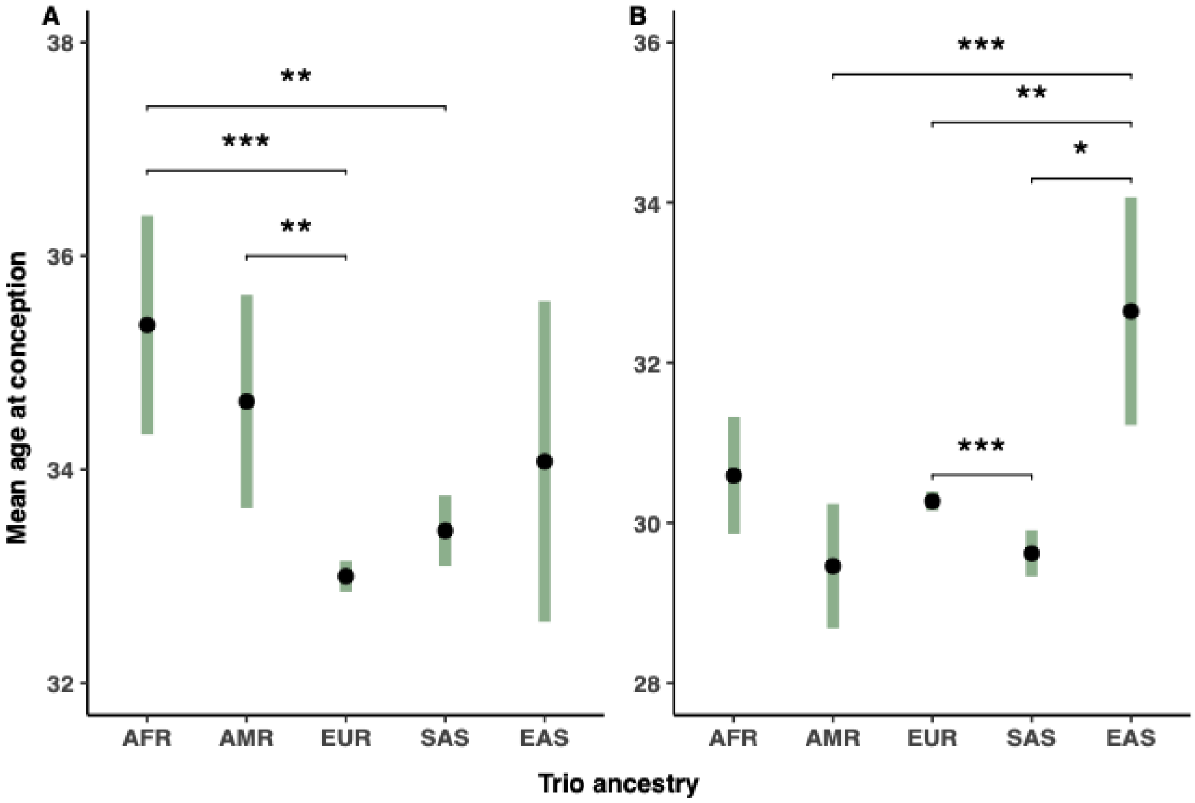


**Supplementary Figure 6. Pairwise parental age at conception differences (sex stratified) across ancestries.** Representing the mean parental age at conception for each ancestry and two-tailed 95% confidence intervals for the mean estimate. **A)** Mean parental age at conception for fathers and **B)** Mean parental age at conception mothers. Asterisks indicate significant differences: ***p <= 0.001, **p<=0.01, *p<=0.05 (ANOVA followed by Tukey HSD test; two-sided p)

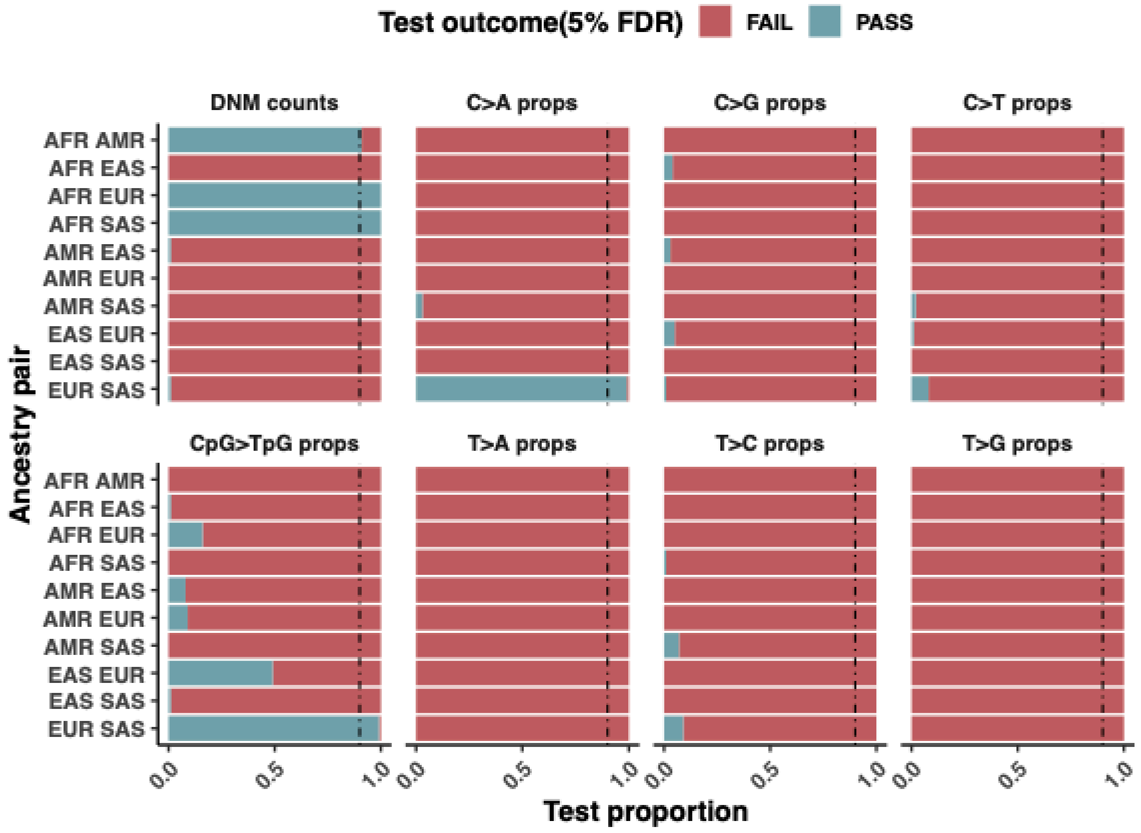


**Supplementary Figure 7. Bootstrapped ancestry associations to DNM counts and spectra over randomly subsampled and age matched trios.** Showing the proportion of association tests passing the 5% FDR threshold for each ancestry pair out of 100 repetitions over randomly subsampled and age-matched data. Panels correspond to either DNM counts (**Model 1** regression), or pyrimidine substitution proportions (**Model 2**) comparisons. The black dotted line intersects 0.9 (i.e. 90/100 repetitions).

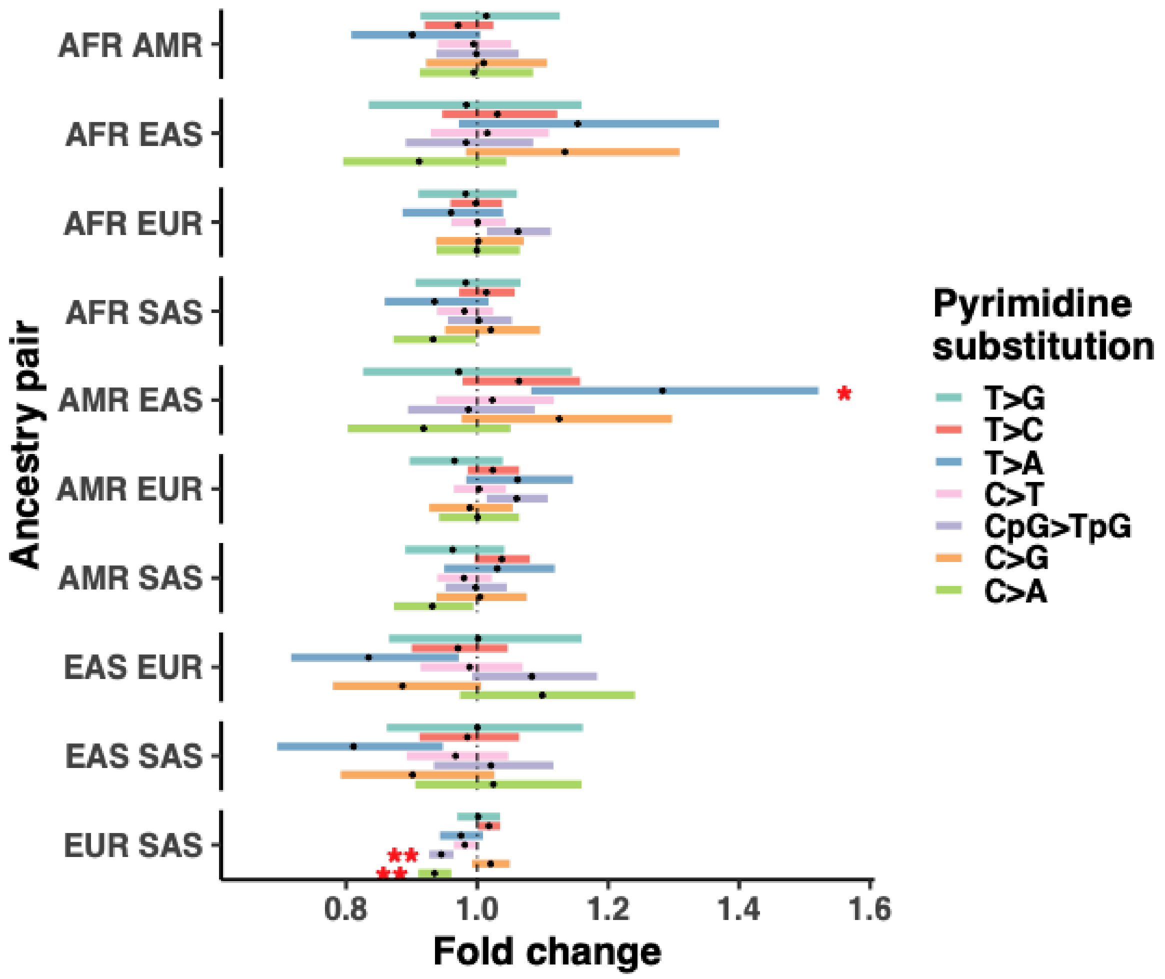


**Supplementary Figure 8. Pairwise comparison of ancestry associated differences on DNM spectra (pyrimidine substitution proportions) per trio** Showing estimated fold change differences on pyrimidine substitution proportions between ancestry pairs (compositional linear regression, **Model 2**, Methods). Effect estimate corresponds to the first ancestry in each row pair relative to the second. Bars correspond to two-tailed 95% confidence intervals for the effect estimate. Asterisks indicate significant associations after multi-testing correction (**5% FDR, *10% FDR) for all non-redundant pairwise tests (n tests = 70).

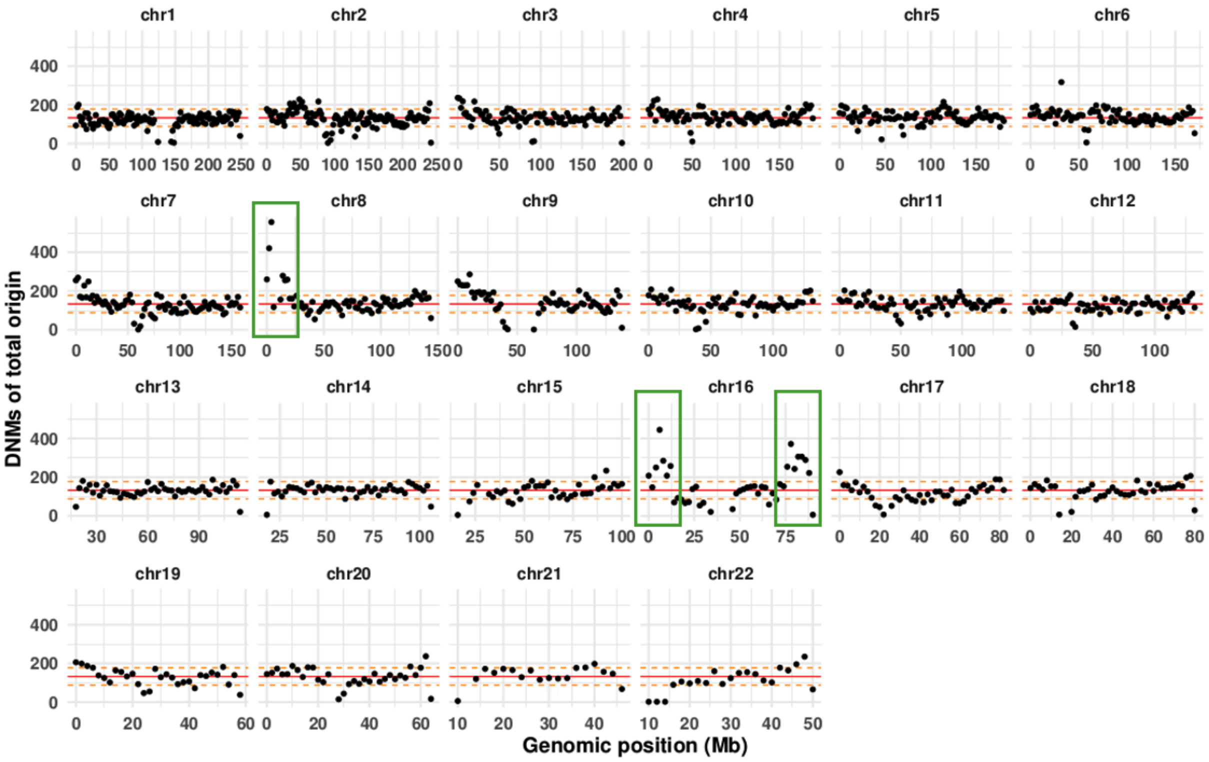


**Supplementary Figure 9. Spatial distribution of GEL unphased DNMs across the human genome (2 Mb binned).** Each point represents the sum of DNMs counts falling into a 2Mb window along a given chromosome. Representing counts per trio (i.e. unphased DNMs). Red line represents the genome-wide DNM count per DNM category (i.e. per trio or parentally phased), while the dotted yellow line represents standard deviation. Excess DNM counts in peri-telomeric regions of chromosomes 8 and 16 are identified with green squares.

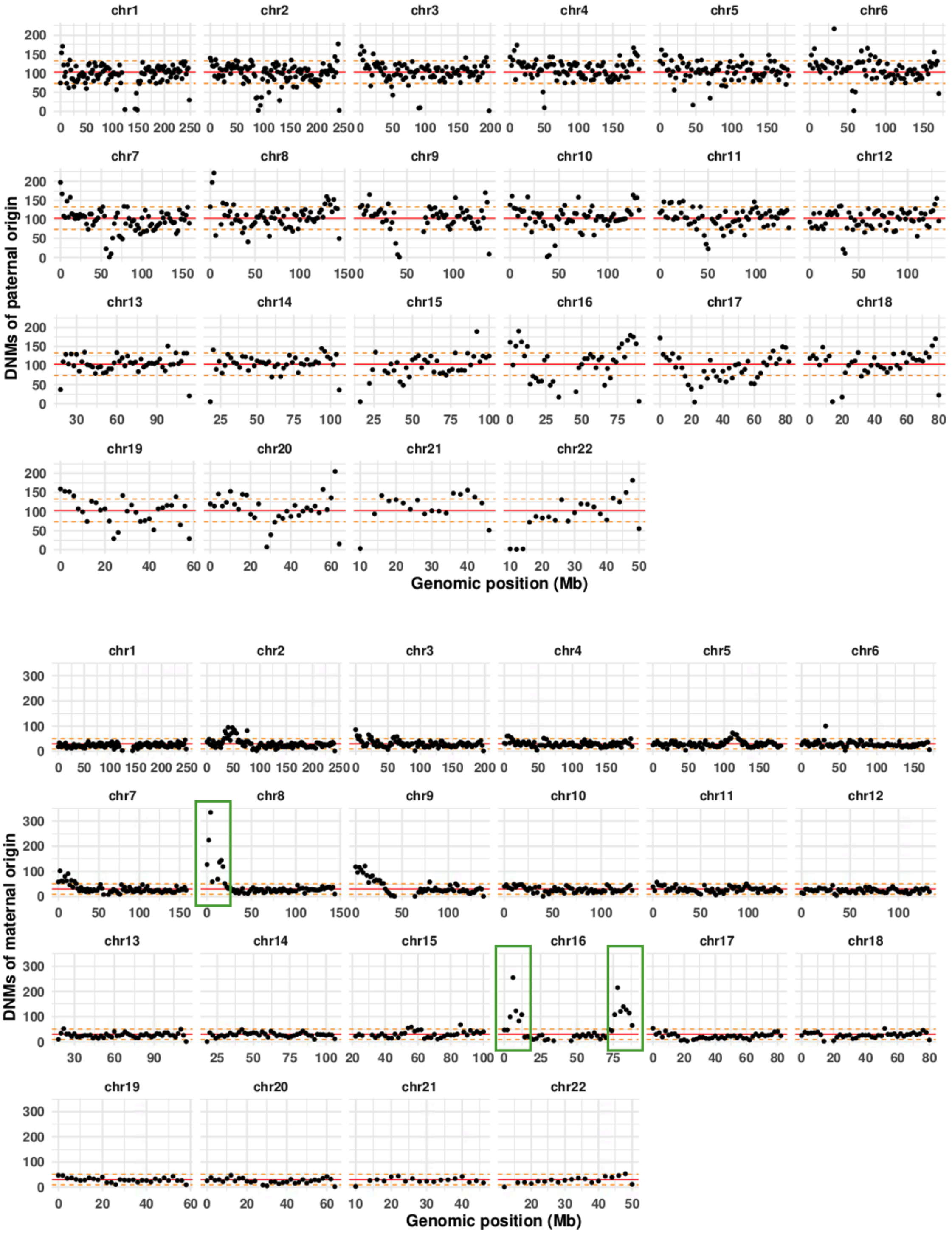


**Supplementary Figure 10. Spatial distribution of GEL phased DNMs across the human genome (2 Mb binned).** Each point represents the sum of DNMs counts falling into a 2Mb window along a given chromosome. Representing counts for paternally phased DNMs (top), and maternally phased DNMs (bottom). Red line represents the genome-wide DNM count per DNM category (i.e. per trio or parentally phased), while the dotted yellow line represents standard deviation. Excess DNM counts in peri-telomeric regions of chromosomes 8 and 16 are identified with green squares.

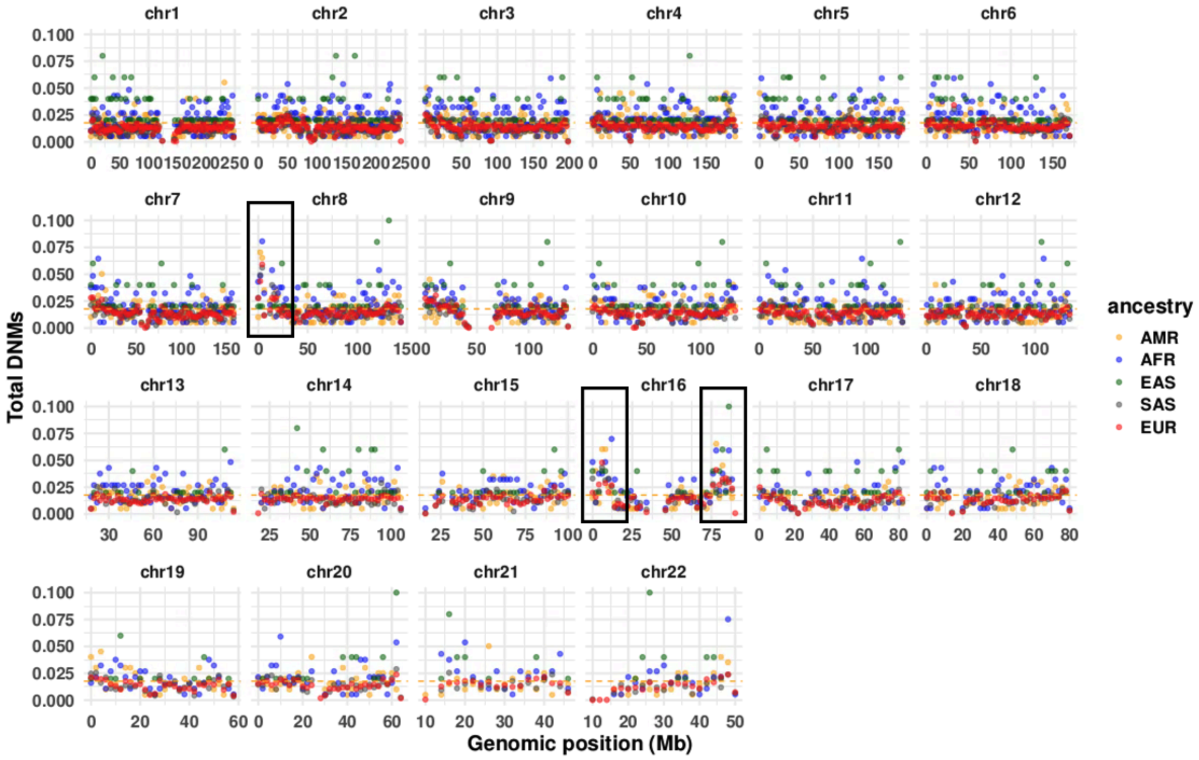


**Supplementary Figure 11. Spatial distribution of ancestry stratified GEL unphased DNMs across the human genome (2 Mb binned).** Each point represents the fraction of phased DNMs counts for that ancestry group falling into a 2Mb window along a given chromosome. Representing counts per trio (i.e. unphased DNMs). Dotted yellow line represents the genome-wide DNM count per DNM category (i.e. per trio or parentally phased). Excess DNM counts in peri-telomeric regions of chromosomes 8 and 16 are identified with black squares.

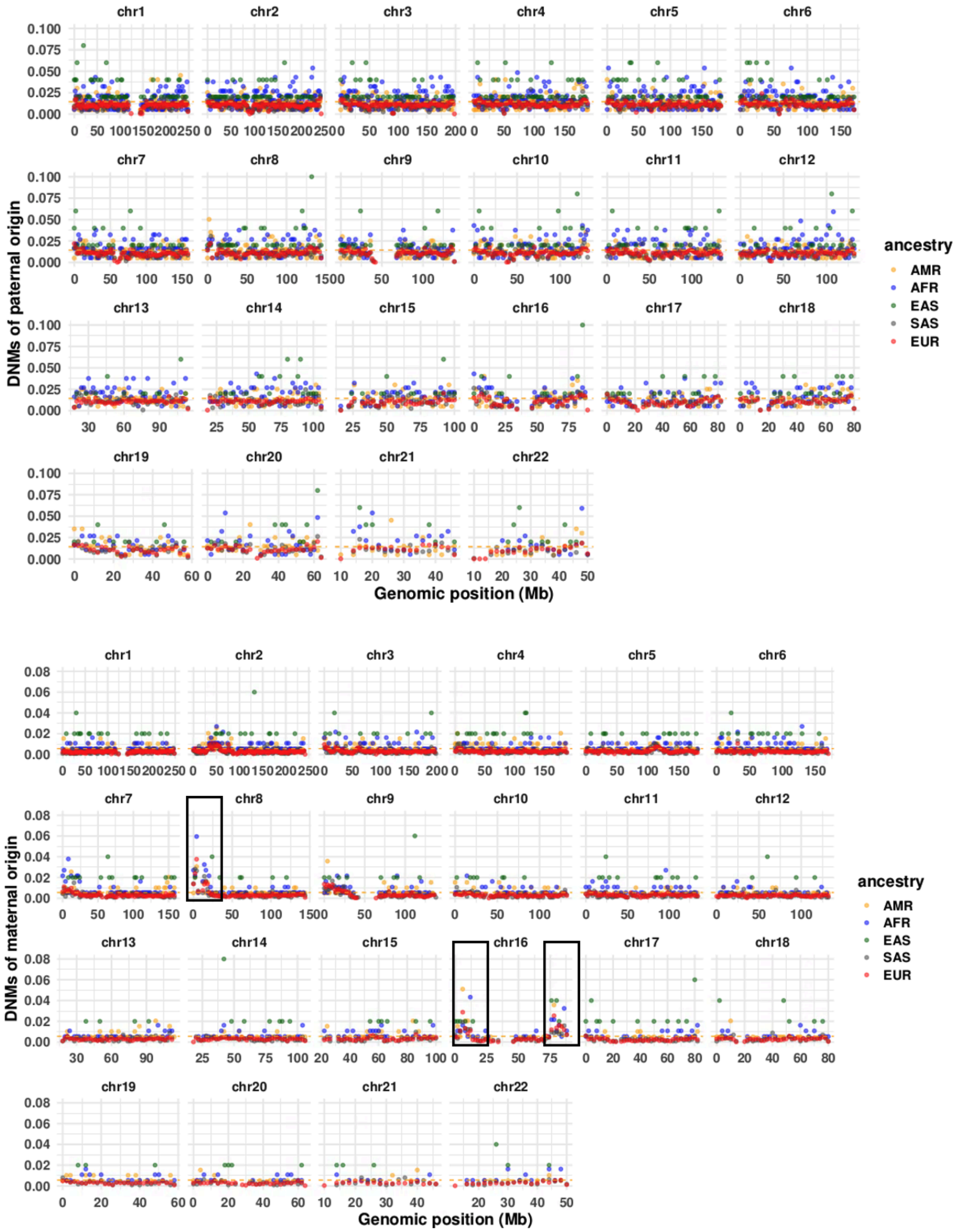


**Supplementary Figure 12. Spatial distribution of ancestry stratified GEL phased DNMs across the human genome (2 Mb binned).** Each point represents the fraction of phased DNMs counts for that ancestry group falling into a 2Mb window along a given chromosome. Representing counts for paternally phased (top), and maternally phased (bottom). Dotted yellow line represents the genome-wide DNM count per DNM category (i.e. per trio or parentally phased). Excess DNM counts in peri-telomeric regions of chromosomes 8 and 16 are identified with black squares

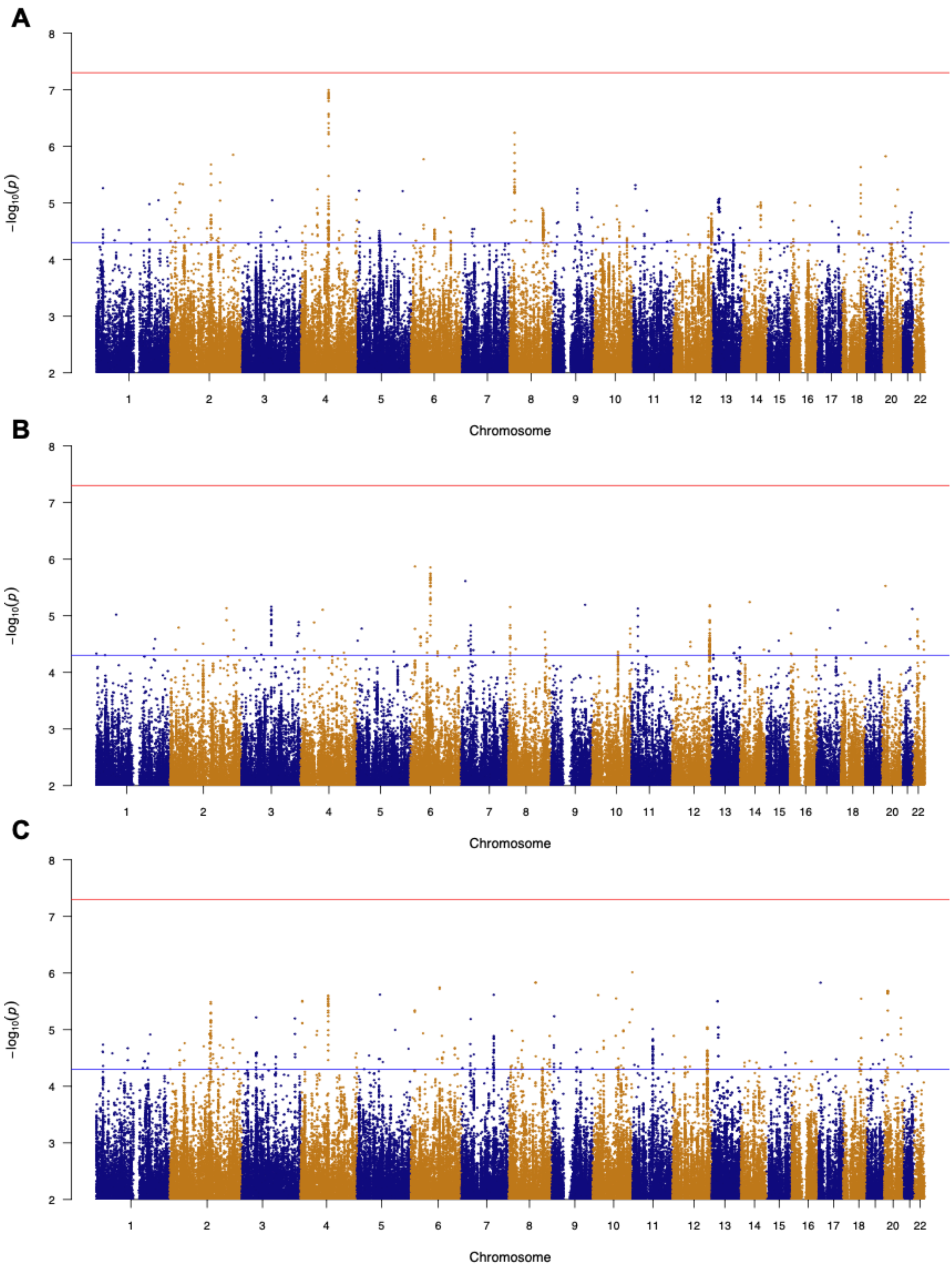


**Supplementary Figure 13. Parental DNM rate GWAS.** GWAS Manhattan plots for individual DNM rate (residualised phased DNM counts per parent, Methods) in the sex-combined model including **A)** both parents **B)**, fathers only, or **C)** mothers only. Blue line corresponds to suggestive significance ($p<=5x10^{-5}$) while the red line corresponds to the genome-wide significance threshold ($p<=5x10^{-8}$).

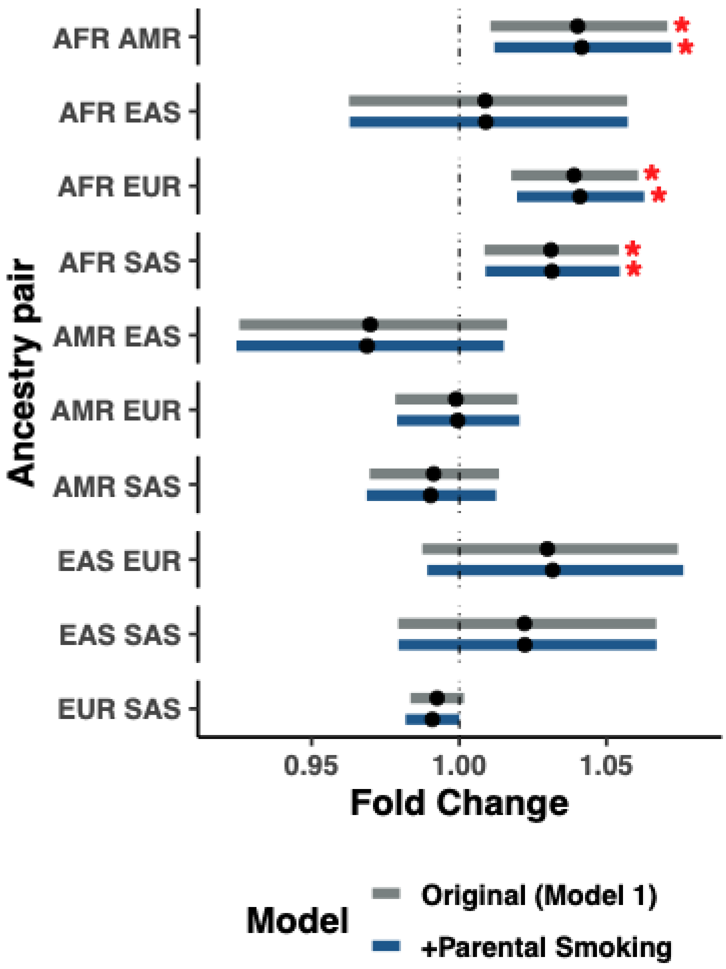


**Supplementary Figure 14. Pairwise comparison of ancestry associated differences on DNM counts per trio before and after controlling for parental smoking behaviour.** Showing fold change difference estimates in DNM counts between ancestry pairs (generalised linear regression, **Model 1**, Methods). The grey bar corresponds to the effect estimate from the original **Model 1** while the blue line represents an independent run of the same model including the parental smoking status per trio (Methods). Effect estimate corresponds to the first ancestry in each row pair relative to the second. Bars correspond to two-tailed 95% confidence intervals for the effect estimate. Asterisks indicate significant fold change differences in DNM counts for the ancestry pair after multi-testing correction in a single experiment (n tests per experiment = 10, 5% FDR).

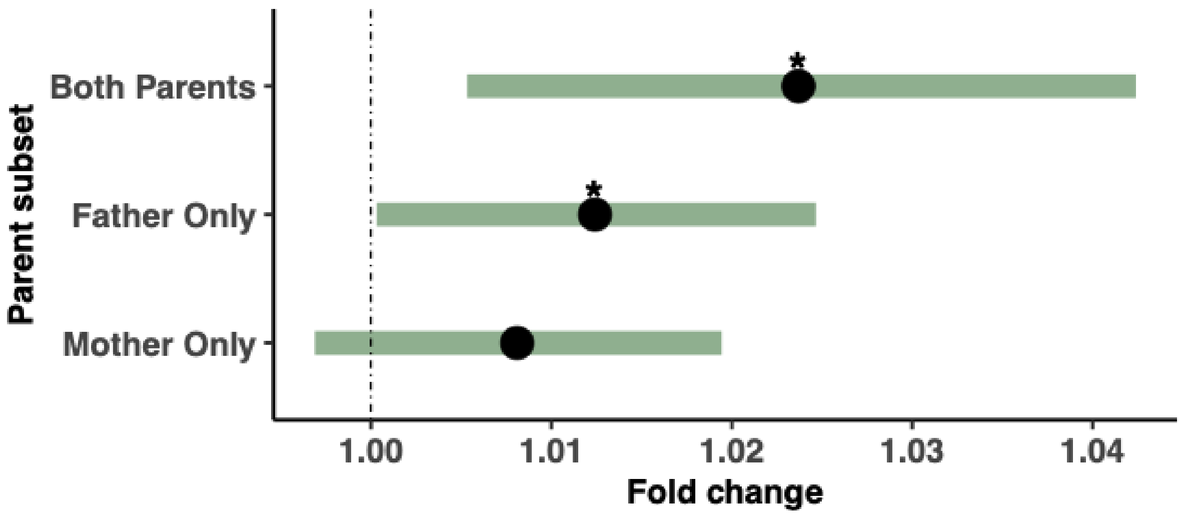


**Supplementary Figure 15. Effect of parental smoking behaviour on DNM rate per trio.** Showing fold change estimates obtained when comparing smoker parent categories (i.e., mother only, father only, both) vs the baseline (i.e., none of the parents smoke). Bars correspond to two-tailed 95% confidence intervals. Asterisks indicate significant fold change differences in DNM counts for the smoker status category (generalised linear regression, nominal p value <= 0.05).

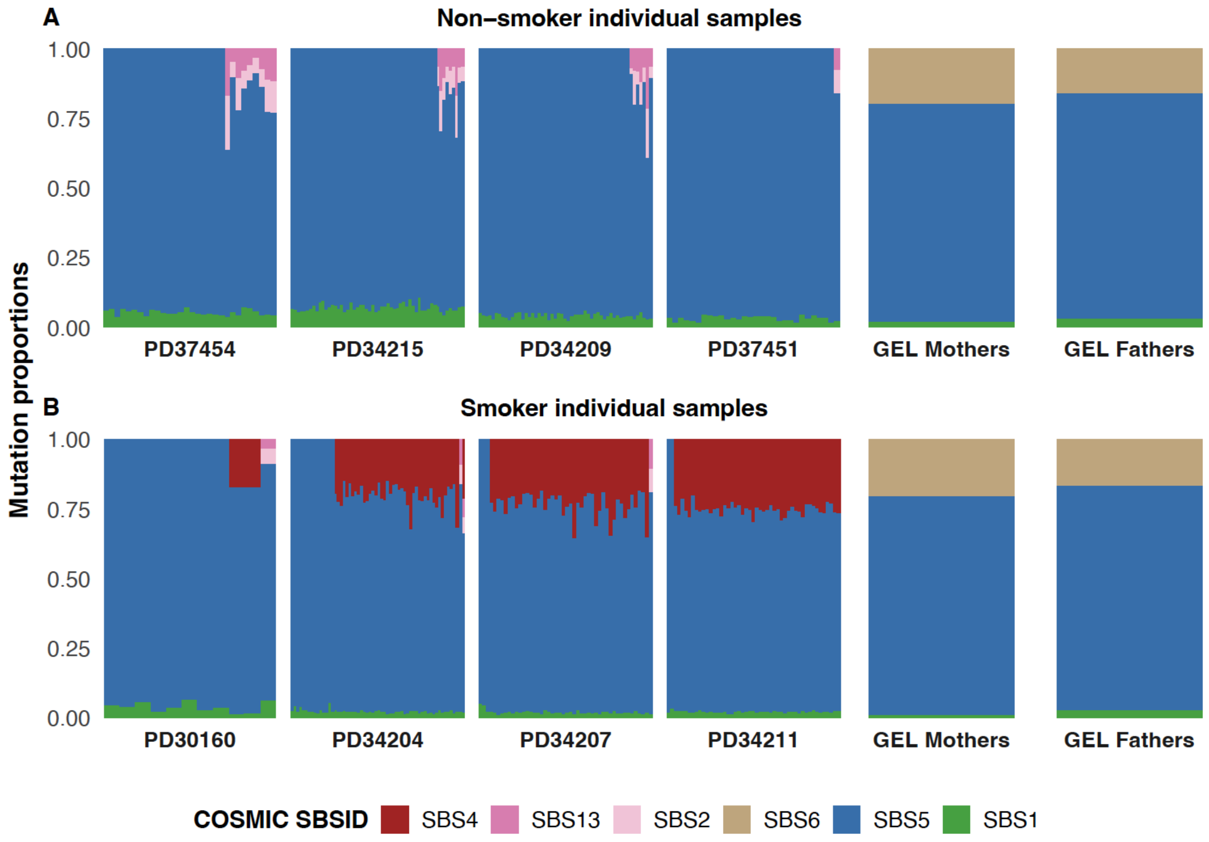


**Supplementary Figure 16. Per-sample mutational signature exposures** For: **A)** non-smoker, and **B)** smoker individuals. GEL individuals represent "meta-individuals" (see **Supplementary Note 4**). Each bar represents the total proportion of mutations assigned to each indicated single base substitution (SBS) signature, amongst somatic mutations detected in bronchial epithelium from four current smokers and four non-smokers from Yoshida et al., 2020 [1], or amongst DNMs from "meta-individuals". Mutation exposures in each sample were deconvoluted into six known mutational processes from the COSMIC v3 catalogue [2] (i.e., SBSIDs).

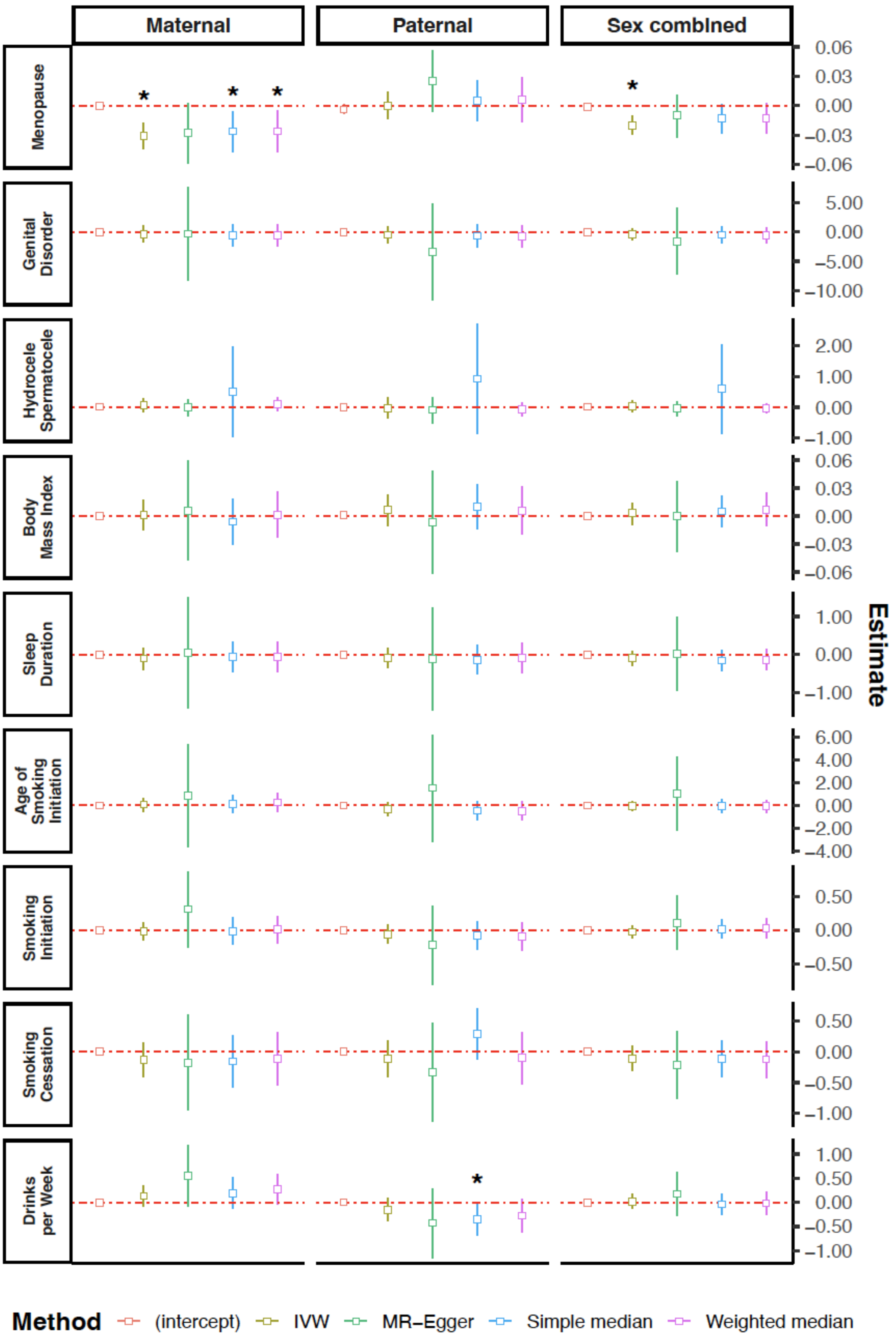


**Supplementary Figure 17. Mendelian randomisation analysis for DNM counts** Forest plots showing estimated causal effects of putative risk factors on phased DNM counts obtained with Mendelian Randomisation. This was conducted on maternally phased DNM counts (left), paternally phased DNM counts (centre), and phased DNM counts in both sexes combined (right). Estimates are computed by four different methodologies (colours): IVW, MR-Egger, Simple median and Weighted median. The intercept from MR Egger is also shown (as a test of directional pleiotropy). Bars correspond to two-tailed 95% confidence

intervals for each estimate. Asterisks indicate nominal statistical significance ($p \leq 0.05$). The exposures considered included: age at natural menopause (“menopause”), diseases of male genital organs (“genital disorder”), sleep duration, hydrocele and spermatocele, smoking initiation (i.e. ever smoked versus never smoked), smoking cessation (i.e. being a current rather than former smoker), age at smoking initiation, drinks per day, and body mass index (BMI). The nominally significant results imply the following directions of effect: later age of menopause causes lower DNM rate in females, and increased number of drinks per week causes a lower DNM rate in males.

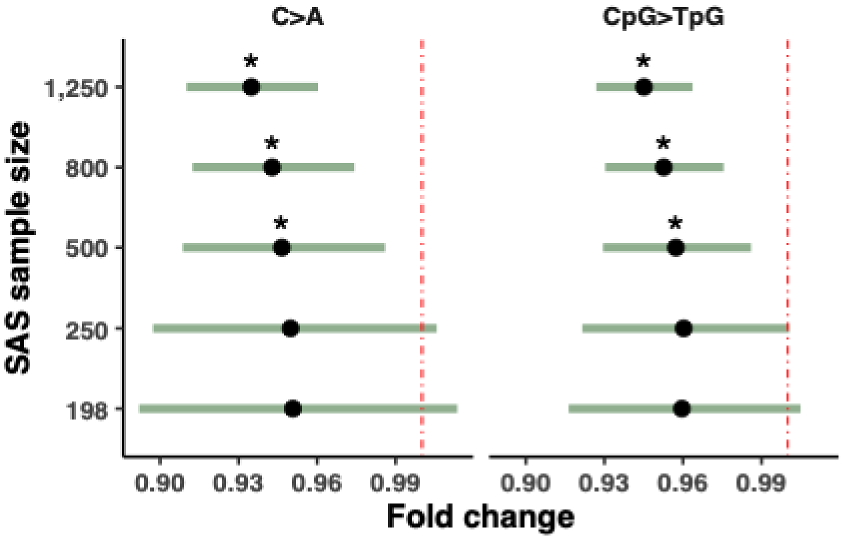


**Supplementary Figure 18. EUR/SAS pair fold change estimate differences for C>A and CpG>TpG proportion across random subsamples of SAS trios.** Fold changes and standard errors represent the mean across 1000 repetitions of **Model 2** run between the EUR group and randomly subsampled SAS trios. Subsample size used in each trial is indicated in the y-axis. The top bar (pink) corresponds to the original fold change estimate difference obtained using the full SAS sample (n=1,250). Bars correspond to two-tailed 95% confidence intervals. Asterisks correspond to significant differences against the null hypothesis of no fold change difference (p<=0.05, Wald test using Z-score from mean estimate and mean standard error).

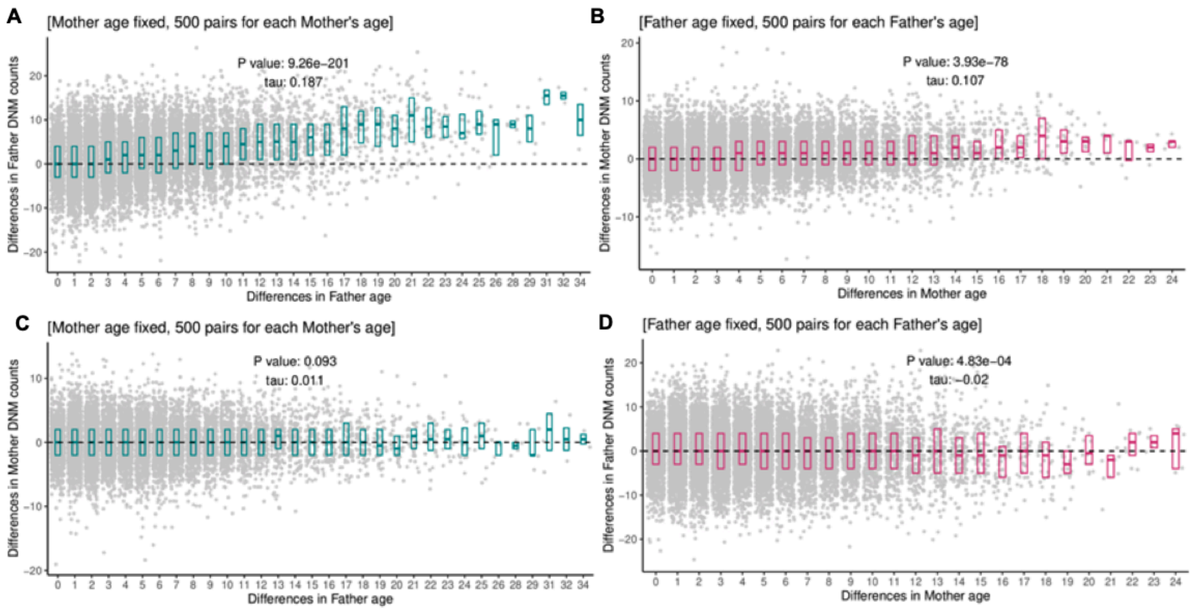


**Supplementary Figure 19. Pairwise comparison of phased DNM counts conditional on the same parental age.** Each point represents a pair of trios, with 500 pairs per age group in each case. The x-axis displays the difference in paternal ages (a), (c) / maternal ages (b), (d) and the y-axis illustrate the difference in paternal (a), (d) or maternal (b), (c) mutation counts. The boxplots represent $25^{th}$, $50^{th}$, and $75^{th}$ percentile. Tau and p-values are evaluated by using Kendall's rank correlation test statistic. The correlations observed in panels (a) and (b) correspond to the expected effects of paternal age on paternal DNMs and maternal age on maternal DNMs respectively. Panel (c) corresponds to an effect of paternal age on maternal DNMs and panel (d) corresponds to an effect of maternal age on paternal DNMs.

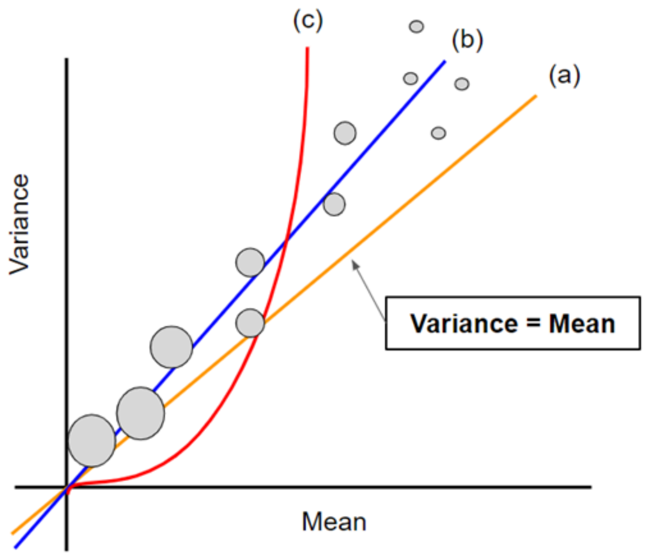


**Supplementary Figure 20. Comparison of the mean-to-variance-relationship between quasi-poisson and other counts distributions.** Illustration of the mean-to-variance relationship of the quasi-poisson distribution (blue line, b). Each circle represents N theoretical samples grouped after binning a continuous variable (e.g. parental age), with radius proportional to N, and are placed in the graph according to the mean and variance of their respective samples. The overall distribution of this theoretical data follows a quasi-Poisson distribution due to the mean-variance overdispersion (i.e. variance exceeds the mean in a linear manner). For contrast, the Poisson (yellow line, a) and negative binomial (red line, c) distributions are also shown.

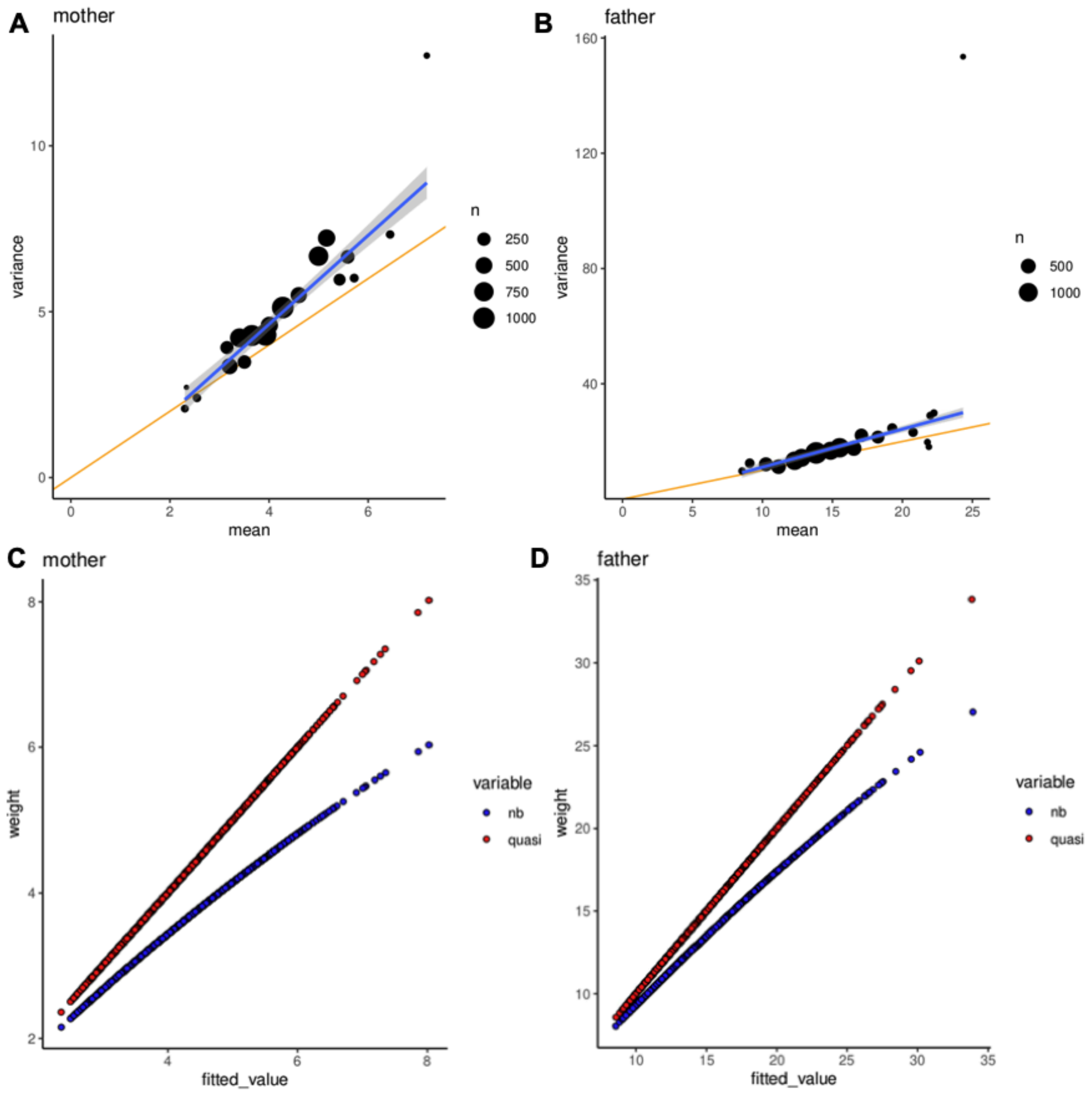


**Supplementary Figure 21. Estimated variance-to-mean relationship for phased DNMs**
**(A-B)** Estimated variance-to-mean relationship for phased DNMs in mothers **(A)** and fathers **(B)**, with a robust regression fit line (blue). The yellow line represents the variance-equals-mean line. The size of the circle represents the number of samples within each binned age group. Note that the rightmost points (which are outliers) contain only a tiny number of samples. **(C-D)** Estimated regression weights as a function of the mean for phased DNMs in mothers **(C)** and fathers **(D)**

# Supplementary Notes

## Supplementary Note 1. Ensuring that the associations between ancestry and DNM counts are not due to technical artefacts or ascertainment bias

### A) Ancestral biases in the reference genome

We wanted to rule out that observed differences in DNM counts between ancestries were due to differences in read mapping quality resulting from ancestral biases in the reference genome. To test this, we first calculated the average number of mismatches of reads containing the alternate allele per DNM (avgNM), and then calculated the mean and maximum avgNM across all DNMs per offspring. We assessed if there were any NM metric differences across all possible ancestry combinations using ANOVA and Tukey HSD post-hoc testing. We found no statistically significant differences between any ancestry pairs (**Supplementary Figure 2**).

We separately included the mean and maximum avgNM values per trio as extra covariates in **Model 1** (main **Methods**) to check whether this significantly altered our original estimates, which it did not (**Supplementary Figure 3**).

Another indicator of potential mapping bias may be the parental coverage for the alternate site classed as "*de novo*" in the offspring. If a given ancestry group has higher mapping errors on average, or is associated with other issues affecting variant calling, variants which are present in the parents and passed on to the child may have low but non-zero variant allele fraction (i.e. fraction of reads carrying the alternate (ALT) allele, VAF), such that the heterozygous parental genotype is not called and these sites are erroneously called as DNMs. To check this, we calculated the mean parental variant allele fraction (VAF) ([maternal VAF + paternal VAF] / 2) at putative DNM sites, calculated the mean across DNMs per offspring, and then included this metric as a covariate in **Model 1** (main **Methods**). We found that this does not affect the original ancestry effect estimates (**Supplementary Figure 3**). This check also implies that our results are unlikely to be driven by the higher rates of heterozygous genotypes in individuals of African ancestries [3]; if heterozygous genotypes in the parents are falsely called as homozygous reference, they are still likely to have low but non-zero VAF, and we have shown that controlling for average parental VAF does not affect our ancestry inferences. We describe additional investigation of this potential artefact below.

### B) Biases due to ancestry-differential rates of post-zygotic mutations

A possible source of false positives DNMs could be post-zygotic mutations (PZMs). This type of mutation would have been acquired during the early embryonic development of the parents and prior to the differentiation of primordial germ cells [4]. Because of this mutation timing, PZMs would become constitutive in the germline and would be mosaic in somatic cells, being present at low VAFs in the parental blood, which would have prevented these variants from being detected during standard variant calling. Although GEL *de novo* variant filtering set a hard filter on parental alternative (ALT) allele coverage of maximum one read in either parent, we sought to test if any DNM with non-zero parental ALT coverage could be a missed PZM, and whether such variants were more common in any particular ancestry

group since if they were, this could potentially be causing a spurious signal of ancestry differences in DNM rates.

Around 13% (n = 73,103) of the DNMs we used in our analyses have a maximum parental ALT coverage of 1 read per parent. By assuming that any of these variants could be a potential PZM, we tested whether these events were more common in any of the ancestry groups we studied. For this, we first classified all DNMs analysed in a given trio as putative PZMs (i.e. ALT read coverage == 1 in either of the parents) or true DNMs (i.e. ALT read coverage == 0 in both parents) and generated counts for these per ancestry. Then, for each ancestry, we asked whether PZM counts were significantly different in that ancestry as compared to the sum of the counts for each event in the rest of the ancestries (i.e. the aggregated counts across all other ancestries). We tested this in a Fisher exact test and found no significant evidence of enrichment (or depletion) of putative PZM events in any of the ancestry groups (**Supplementary Figure 4**).

### C) Biases due to ancestrally-differential rates of missed constitutive heterozygous calls in parents

As noted above, another possible source of false positive DNM calls could be missed constitutive heterozygous genotypes (HETs) in the parents. As the number of HET genotypes is a function of genetic diversity, this is known to vary across continental ancestry groups, and to be the highest among AFR descendants [3]. Hence, if the GEL DNM calling and filtering pipeline systematically under-called HETs, the AFR group would be most affected by this effect and would in turn display an artifactually increased number of DNMs when compared to other ancestries.

To test this hypothesis, we first compared the distribution of parental VAF of DNMs detected in AFR probands to that of DNMs detected in other probands. If AFR DNMs had an increased HET contamination, its median parental VAF at these DNM sites would be significantly higher. We tested this hypothesis using a Kolmogorov-Smirnoff test, and found it was non-significant (p value = 1). We obtained similar results when comparing the mean parental VAF across all possible ancestry pairs using ANOVA and Tukey HSD post hoc testing (all adjusted p values > 0.05).

Similarly, when restricting the analysis to DNMs with parental ALT allele coverage > 0 (n = 73,103), the comparison of parental VAF distribution between AFR and non-AFR DNMs still showed no significant differences **(Supplementary Figure 5A**; Kolmogorov-Smirnoff test p value = 0.65). On the other hand, the pairwise comparison of mean parental VAFs showed subtle but significant differences between the mean parental VAF of SAS/EUR and SAS/AMR ancestry pairs (**Supplementary Figure 5B**, ANOVA/Tukey HSD post hoc test adjusted p value <= 0.05). However, these were not pairs for which we found significant DNM count differences (**Figure 1A**). Next, we tested the effects of removing DNMs with parental ALT allele coverage > 0 from the pool of DNMs used to run Model 1. This resulted in slightly wider confidence intervals for the AFR/SAS and AFR/AMR comparisons, which reached nominal significance ($p < 0.05$) but did not pass multi-testing correction (**Supplementary Figure 5C**; FDR > 5%).

If under-calling of parental heterozygous sites were leading to inflated DNM counts in populations with higher heterozygosity, we would expect to see an association between the number of heterozygous sites in parents and the number of DNMs in their offspring within a given ancestry group, since the number of uncalled heterozygous sites is likely to be proportional to the number that have been called and passed QC. To test this, we calculated the number HETs per parent for all trios used in our original DNM ancestry regression (n = 9,820). We calculated HET counts from variants called at the individual level that passed basic sample-level quality filters described elsewhere (https://re-docs.genomicsengland.co.uk/sample_qc/).

We then tested the association between the number of DNMs per trio and the mean number of HETs per parent while stratifying by ancestry. To do this, we fitted a generalised linear regression model of the quasipoisson family in each ancestry group separately, while controlling for technical and biological covariates associated with the number of DNMs per trio as shown in the following model (**Supplementary Model 1):**

$$\begin{gathered} trio\ DNM\ counts\ = \beta_0\ +\ mean\ parental\ nHETs\ \bullet\ \beta_1\ + \\ maternal\ age\ at\ conception\ \bullet\ \beta_2\ +\ paternal\ age\ at\ conception\ \bullet\ \beta_3\ + \\ mean\ sequencing\ depth_{\ mother}\ \bullet\ \beta_4\ + mean\ sequencing\ depth_{\ father}\ \bullet\ \beta_5\ + \\ mean\ sequencing\ depth_{\ offspring}\ \bullet\ \beta_6\ + \\ percent\ aligned\ reads_{\ mother}\ \bullet\ \beta_7\ + percent\ aligned\ reads_{\ father}\ \bullet\ \beta_8\ + \\ percent\ aligned\ reads_{\ offspring}\ \bullet\ \beta_9\ + \\ median\ Bayes\ Factor\ \ per\ trio\ \bullet\ \beta_{10}\ +\ median\ VAF\ per\ trio\ \bullet\ \beta_{11}\ +\ \varepsilon \end{gathered}$$

where the $mean\ parental\ nHETs$ represent the average number of HETs across both parents in a given trio. The rest of the covariates are the same as those outlined in Model 1 in the main Methods section.

We found that the mean parental nHETs were not associated with DNM counts in any of the ancestry groups (**Supplementary Data 8**; p value > 0.05). Although the heterozygosity between parents of the same ancestry is expected to be similar, we also tested whether the number of HET sites in the maternal and paternal genomes contributed independently to the DNM counts per trio by including these metrics in the **Supplementary Model 1** instead of the mean parental nHET. We found no significant evidence of association between DNMs and either nHET metric (**Supplementary Data 8**; p value > 0.05).

Altogether, the results from these analyses suggest that the increased number of DNMs in the AFR group is unlikely to be driven by an excess of missed heterozygous variants in the parents.

**D) Ancestry-related ascertainment biases into the dataset**

It is conceivable that there may have been ascertainment biases during recruitment to the 100,000 Genomes Project, such that families from certain ancestry groups were more likely to be recruited if their affected child had a pathogenic DNM rather than some other genetic or non-genetic cause. This would be expected to manifest in differences in the number of deleterious DNMs (most of which are probably protein-altering) between ancestries, which

could, in theory, drive the ancestral differences we see in overall DNM rate. We counted the number of protein-coding DNMs per proband (including those with worse consequence *"missense_*", "start_lost", "stop_lost", "stop_gained", "stop_retained",* or *"splice_*"*) and included this as an extra covariate in **Model 1** (main **Methods**). The observed ancestry associations to DNM rate remained unchanged, suggesting that these were not due to ancestry-related ascertainment biases (**Supplementary Figure 3**).

### E) Biases due to differences in parental age between ancestry groups

Although our main analyses did control for parental age, we wanted to be sure that differences in parental age distribution across ancestries are not driving any of our reported association signals. Evidence from genetic variation has shown that the generation interval time (i.e. parental age at conception) has varied across different human populations throughout human history [5]. It is also well described that, in modern times, societal and economic factors influence parental age at conception across continental populations [6].

Such differences are also detected in the Genomics England *de novo* mutation trio cohort parents included in this study (**Supplementary Figure 6**). Here, pairwise ancestry testing showed that the mean difference in maternal age at conception is statistically significant between the AMR-EAS, EAS-EUR, EAS-SAS, and EUR-SAS pairs (ANOVA and Tukey post hoc testing, p adjusted <= 0.05). On the other hand, the mean difference in paternal age at conception is significantly significant between the AFR-EUR, AFR-SAS and AMR-EUR pairs (ANOVA and Tukey post hoc testing, p adjusted <= 0.05). Some of the population pairs that showed significant differences in parental age were also those that showed significant differences in DNM counts or spectra (AFR-EUR, AFR-SAS and EUR-SAS). In these cases it is plausible that our detected differences in DNM count or pyrimidine proportion differences could have been driven by systematic differences in parental ages across the relevant ancestry pairs. Although we note that we include the maternal and paternal ages at conception as independent covariates in our regression models (**Model 1** and **Model 2**), we tested the robustness of our associations through subsampling and permutation strategies.

First we subsampled each ancestry to approximate the mean parental age from a reference ancestry group (e.g. the mean paternal age from the AFR group). For this, and for each non-reference ancestry group, we randomly selected up to 75% of trios (i.e. the working subsample), and kept the remaining trios (i.e. the balancing subsample) for resampling as follows. From the balancing subsample, we iteratively sampled N individuals (without replacement), and used them to replace elements in the working subsample until the mean of the working subsample was <0.3 years of the mean of the reference ancestry group. The goal of this was to 1) approximate the target mean while keeping as many trios as possible (to maintain statistical power), and 2) ensure no significant parental age differences between the subsampled and reference ancestry (which we corroborated based on Kolmogorov Smirnoff and ANOVA-TukeyHSD tests).

Overall, we performed two main subsampling experiments using the method above. First, due to the significant differences in paternal age in the AFR-SAS and AFR-EUR comparisons, we subsampled all ancestry groups to match the mean paternal age from the AFR group to re-run **Model 1** (i.e. DNM count associations), in which we had seen significantly elevated DNM counts in the AFR group. Secondly, due to the significant

difference in maternal age in the EUR-SAS comparison, we subsampled all ancestry groups to match the mean maternal age from the SAS group to re-run **Model 2** (i.e. DNM spectra associations), in which we had seen significant spectra differences between SAS and EUR. We repeated each of these subsampling experiments 100 times, each time running **Model 1** and **Model 2** on the relevant subsample, and counted the number of times we found a statistically significant association at 5% FDR for a given ancestry pair. We found that the associations obtained with the full cohort are replicated across >=90% of the age matched repetitions (**Supplementary Figure 7**), thus concluding that paternal and maternal age differences across ancestries are not driving any of our reported association signals.

## Supplementary Note 2. Comparing ancestry-associated differences on mutation spectra using DNM data and polymorphism data

Ancestry-associated differences in germline mutation spectra were previously reported by Harris and Pritchard [7]. These were discovered using common polymorphism data from the 1000 Genomes Project. Briefly, each SNP in that dataset was classified according to its ancestral and derived allele (C>A, C>T, C>G, A>T, A>G, A>C), and the base pair immediately 5' and 3' of it [7]. This results in each SNP being classified into 1 of 96 possible combinations of flanking context and ancestral-to-derived allele substitutions. Counts for each of the 96-substitutions were generated for each of the five continental super-populations in the 1000 Genomes Project (AFR, AMR, EAS, EUR, SAS). Ancestry-associated differences in spectra were calculated as follows. First, for a given substitution (e.g. “A[C>T]A”), population-specific proportions were calculated as the ratio between the substitution of interest over the sum of the counts for the rest of the substitutions in that population. Then, for each substitution category, population-specific proportions in two populations of interest (e.g. EUR and SAS) were compared.

We compared our mutational spectra results with the findings described by Harris and Pritchard. For this, we obtained the supplementary data corresponding to the Supplementary Figure 1 of their paper [7], which consisted of a 5x96 count matrix (ancestry x substitution category). We changed the encoding of the 96-substitution code from this data to make it compatible with our own annotations. Due to limited power in our study, we were not able to consider the full 96-substitution code, so we collapsed counts in the 96-substitution code presented in their paper into a 6-pyrimidine substitution code (switching the substitution to the complementary strand where necessary so we considered only C>T, C>A, C>G, T>A, T>G, and T>C variants). Finally, to obtain p-values for each ancestry comparison and pyrimidine substitution, we followed the same method described by Harris and Pritchard. Briefly, we first obtained chi-square p-values for all population-pairs and substitution comparisons using 2*2 count matrices as shown below:

| | |
|---|---|
| $S_{p1}^{(m)}$ | $T_{p1} - S_{p1}^{(m)}$ |
| $S_{p2}^{(m)}$ | $T_{p2} - S_{p2}^{(m)}$ |

where $S_{px}^{(m)}$ represents the number of substitutions of type $m$ in population $x$, and $T_{px}$ represents the total number of substitutions in that population.

Then, for each population pair, we obtained ordered p-values by iteratively comparing counts for the substitution with lowest p-value against the counts of the substitution with the next lowest p-value as illustrated below.

| | |
|---|---|
| $S_1^{(m_i)}$ | $\Sigma^{n_{pyr\,subs}}_{j=i+1} S_1^{(m_j)}$ |
| $S_2^{(m_i)}$ | $\Sigma^{n_{pyr\,subs}}_{j=i+1} S_2^{(m_j)}$ |

where $m_i$ represents the substitution with the lowest p value, $m_j$ represents the substitution for the next lowest p-value, and $n_{pyr\,subs}$ represents the size of the substitution code being tested (e.g 6 pyrimidine substitutions). As in Harris' work, all comparisons with an ordered p-value <= $1x10^{-5}$ were deemed to be significant.

We compared the results from Harris and Pritchard against the significant spectrum changes that we identified in our study (main **Figure 1B**), specifically for SAS and EUR pairs, for which we identified two significant associations. Harris and Pritchard data showed a significant enrichment of C>A (Fold Change [FC] = 1.011, ordered p = $2.21x10^{-20}$), and depletions of T>C (FC = 0.994, ordered p = $3.58x10^{-5}$), and T>G (FC  = 0.986, ordered p = $1.05x10^{-25}$) substitutions (**Supplementary Data 3**). Aiming to account for the differential mutation rate produced by the spontaneous cytosine deamination occurring in CpG islands [8], we defined the category CpG>TpG for C>T sites occurring at  CpG sites. We found that CpG>TpG proportions were enriched in SAS compared to EUR (FC = 1.020, ordered p = $2.29x10^{-70}$; **Supplementary Data 3**). We note that the effect directions in these comparisons are inverted compared to ours since we used SAS ancestry as the baseline. Hence, the differences reported by Harris and Pritchard would be equal to the inverse of the fold change (i.e. 1/FC) when using the SAS ancestry as the baseline. Taking this into account, and by inverting the Harris & Pritchard FC effect directions to match our EUR vs SAS comparisons, our study recapitulated 2/4 of their associations (FDR<=5%), namely C>A (Harris & Pritchard 1/FC = 0.98; GEL FC = 0.93), and CpG>TpG (Harris & Pritchard 1/FC = 0.98; GEL FC = 0.94).

## Supplementary Note 3. Checking ancestry specific DNM spatial distribution in the human genome

Following a strategy used in a published work [9], we divided the genome into 2Mb windows and then calculated the number of DNMs in each bin. We corroborated that we were able to replicate previous findings [9] regarding an increased number of DNMs occurring in the peri-telomeric regions of chr8 and chr16 (**Supplementary Figure 9**), which are mostly attributable to maternal DNMs (**Supplementary Figure 10**). We then reproduced this analysis while stratifying by ancestry. As raw counts per bin would reflect the sample size imbalance of this cohort (dominated by the EUR group), we normalised DNM counts per bin per ancestry by the total DNMs detected in a given ancestry and phasing group (**Supplementary Figure 11,12**). This visualisation reproduces the observations on maternal DNM excess in the chromosome 8 and 16 peri-telomeric regions, which seem consistent across all ancestry groups (**Supplementary Figure 12**). Ancestry-specific spatial clustering signals are difficult to ascertain due to the limited sample size for most non-EUR groups. In particular, it is difficult to assess the significance of individual peaks (e.g. total DNMs in chr22, **Supplementary Figure 11**), but the fact that none are apparent in the best-sampled ancestry group (EUR) suggests that more data would be necessary to confidently investigate these signals. We also note that very low DNM counts in windows next to centromeres and telomeres very likely reflect DNM callability in these regions.

## Supplementary Note 4. Attempting to identify associations between parental smoking behaviour and DNM mutation spectra

We first tested if smoking is a significant predictor of pyrimidine substitution proportion differences in parentally phased DNMs. For this, in the same way to other analyses, we annotated parentally phased DNMs according to their pyrimidine substitution type (i.e. C>A, C>G, CpG>TpG, C>T, T>A, T>C, T>G) and calculated the proportion of DNMs in each substitution group per individual (out of total phased DNMs per individual). Then, we regressed each pyrimidine proportion on the smoking status of the individual, parental age at conception, and different quality control covariates, as shown in the next model (**Supplementary Model 2**):

$$\begin{gathered} phased\,pyr_y\ proportion\ = \beta_0 + ever\ smoked_{(0|1)} \bullet \beta_1 + \\ parental\ age\ at\ conception \bullet \beta_2 + \\ mean\ sequencing\ depth_{mother} \bullet \beta_3 + mean\ sequencing\ depth_{father} \bullet \beta_4 + \\ mean\ sequencing\ depth_{offspring} \bullet \beta_5 + \\ percent\ aligned\ reads_{mother} \bullet \beta_6 + percent\ aligned\ reads_{father} \bullet \beta_7 + \\ percent\ aligned\ reads_{offspring} \bullet \beta_8 + \\ total\ SNVs_{mother} \bullet \beta_9 + total\ SNVs_{father} \bullet \beta_{10} + total\ SNVs_{offspring} \bullet \beta_{11} + \\ median\ Bayes\ Factor\ per\ trio \bullet \beta_{12} + median\ VAF\ per\ trio \bullet \beta_{13} + \varepsilon \end{gathered}$$

where $ever\ smoked$ was derived in the same way as described in the main methods. We restricted this analysis to individuals with DNM phase and EHR data available (n fathers = 6,599 fathers; n mothers = 9,133), and ran regression using the compositional regression method (linDA) described by Zhou et al., 2022 [10]. We did not find any significant association between smoking behavior (smoker [1] versus non-smoker [0]) and any of the tested pyrimidine substitution proportions after accounting for multiple testing (5% FDR, n tests = 7, **Supplementary Data 9**).

Next, we asked if known mutational signatures associated with smoking behaviour could be deconvoluted from phased DNMs obtained from parents who smoke. Different somatic mutational processes operating across tissues display distinctive mutational patterns that can be deconvoluted using dimensionality reduction and classification algorithms [11]. Broadly, this process consists of the steps: 1) generation of mutation counts matrices per sample, with mutations typically annotated using a 96-pyrimidine substitution code [12,13], 2) *de novo* signature extraction, which aims to identify single base substitution (SBS) patterns in the input data in an unbiased manner [13], and 3) signature decomposition, which aims to compare the identified *de novo* patterns to publicly available annotated SBS signatures, such as the COSMIC database [2,13].

To apply these methods to our own data, we first annotated all parentally phased DNMs according to 1) their pyrimidine substitution direction, and 2) their flanking 5' and 3' base pair, after which each DNM was classified in 1 of 96 possible SBSs. As mutational signature extraction is a process usually applied to somatic samples (with a mutation load several

orders of magnitude higher than that of the germline [14]), we reasoned that the average number of phased DNMs in a single individual (~15 for fathers, ~4 for mothers) may be insufficient to accurately identify mutational signatures. For this reason we pooled together phased DNMs by smoking status group and sex to create four “meta-individuals”, each representing all of: 1) smoker fathers, 2) smoker mothers, 3) non-smoker fathers, and 4) non-smoker mothers. Then, for each of these synthetic samples, we counted the occurrence of each 96 SBSs. Ninety-nine DNM sites were shared by more than one individual in a given pool, and we removed duplicate variants from each meta-individual before counting. To prevent potential ancestry-related noise, only EUR individuals were considered for DNM pooling. The total number of DNMs and individuals represented by each meta-individual is shown in **Supplementary Data 10**. Aiming to increase our power to pick up any smoking signature signal, we merged our count matrix with counts obtained from an external reference panel containing 337 normal lung (noncancerous bronchial epithelium) samples from four ascertained smokers and four non-smokers [1]. This matrix was used to identify *de novo* mutational signatures using HDP [15,16]. The identified *de novo* signatures were compared against the COSMIC SBS v3 database [2,12] using the cosine similarity metric implemented in the “lsa” R package [17]. We kept all *de novo* signatures / COSMIC SBS pairs with cosine similarities >= 0.8, or containing any of the COSMIC signatures reported by Yoshida et al., 2020 [1] for the included external samples. With this, we deconvoluted *de novo* signatures into 6 COSMIC SBS signatures using the “*decompose.fit*” function implemented in the SigProfilerExtractor software [13]. Although we were able to correctly identify the hallmark tobacco smoking SBS signature (COSMIC’s SBS4) in the external smoker individuals, we did not identify this in any of the meta-individuals from GEL (**Supplementary Figure 16**).

There could be several reasons behind the apparent absence of any smoking-associated signatures in the GEL smokers. First, we may just be underpowered to detect this since the number of DNMs in our smoker “meta-individuals” is, on average, more than an order of magnitude lower than the average number of somatic mutations in the lung tissue from smokers sequenced in Yoshida et al. (**Supplementary Data 10**). Second, even though it is well established that tobacco smoke is causal for specific mutational signatures in lung tissue [1], its effect may not be the same across other tissues, including the germline. On this note, even lung tissue in regular smokers displays some degree of heterogeneity in terms of mutation burden and overall tobacco smoke signature load [1]. Such heterogeneity may also be expected from tissues not directly exposed to tobacco smoke, potentially even to a higher degree. As it has been shown that tobacco smoke signatures are present in cancerous tissue outside the lung, such as bladder cancers [18], we cannot rule out that smoking-associated signatures can be found outside of the lung (even in the germline), but a larger sample size may be required to detect this signal.

## Supplementary Note 5. Estimating power to detect spectra differences in GEL DNMs as compared to polymorphism data

Harris and Pritchard (2017) [7] analyses on polymorphism data report numerous significant differences in mutation spectra across genetically inferred ancestry groups. However, our work revealed only two significant associations (FDR<=5%), namely C>A or CpG>TpG between the EUR and SAS groups (**main Figure 1**). Our lack of associations between the other groups of smaller sample size could have been due to the reduced sample size of these groups as compared to those of EUR (n= 1,250) and SAS origin (n= 8,104 and 1,250, respectively). We conducted the following analyses to assess this possibility.

First, we re-evaluated the significant DNM spectra differences detected in our analysis (FDR ≤ 5%). We specifically focused on C>A and CpG>TpG substitutions between EUR and SAS groups, for which we observed a mean fold change of 0.93 (i.e. 6.51% absolute change). To investigate the impact of sample size on our ability to detect a significant effect, we performed a downsampling experiment by randomly subsampling the SAS group (n = 1,250) to sizes of 198 (the sample size of the AFR group in our data), 250, 500, and 800 trios. For each subset, we repeated the DNM spectra associations using **Model 2**, performing 1,000 iterations at each sample size. We then averaged the log2 fold changes and standard errors across these iterations for each pyrimidine substitution. Our results showed that downsampling the SAS group to 198 trios (matching the sample size of the AFR group) did not produce any significant fold change estimates for C>A or CpG>TpG substitutions (i.e. the fold changes were not significantly different from the null expectation of 1). Furthermore, we determined that a significant fold change for these substitution types was only observed with a sample size >500 SAS trios (**Supplementary Figure 18**).

We then looked at the effect sizes observed in polymorphism data from Harris and Pritchard, (**Supplementary Data 3**). In this data, the strongest fold change is 0.956 (i.e. 4.3% absolute change) for C>T substitutions between the equivalent EAS and EUR groups in that study. Taking into account that in our DNM data a sample size of >250 SAS trios was necessary to detect a significant fold change that is higher than the strongest effect observed in polymorphism data (i.e. ~4.3% change), this suggests that if the mutagenic factors affecting polymorphisms are still active to the same extent in contemporary populations, we may have failed to detect them due to the sample sizes of particular ancestries (n AFR = 198, n AMR = 215, n EAS =53). Note however that a formal power analysis would require simulation with multiple mutational and ancestry-specific parameters that are currently unknown.

## Supplementary Note 6. Cross-parental effects on early embryonic mutations

It has been suggested that maternal ageing may lead to an increased frequency of early post-zygotic mutations during the initial cell divisions of the embryo [19]. Some of these mutations may then appear in some or all offspring somatic cells and be called DNMs. In particular, those occurring on paternally derived chromosomes would be interpreted as paternal DNMs, meaning that such an effect could manifest as an effect of maternal age on the number of paternally DNMs. Gao et al.[19] found just such a signal in a dataset of 1,548 Icelandic trios [9]. Replicating their analysis, we investigated within- and cross-parental effects in our much larger cohort, as shown in **Supplementary Figure 19**.

Panel (a) shows the effect of paternal age on paternal mutations, controlling for maternal age. Each point represents a pair of trios in which the maternal age is the same, and where the difference in paternal ages between the trios represented by the x-coordinate and the corresponding difference in phased paternal DNM counts is represented by the y-coordinate. For each maternal age in the dataset, 500 trio pairs were selected at random. As expected, this plot shows an increased differential paternal DNM count with increasing difference in paternal age, corresponding to the well-established effect of paternal age on paternal DNMs. Panel (b) shows the equivalent within-parental effect of maternal age on maternal DNMs, here plotting trio pairs with the same paternal age, for all paternal ages in the dataset.

Panel (c) shows the effect of paternal age on maternal mutations. Here as in panel (a), each point represents a pair of trios in which the maternal age is the same and the difference in paternal ages is represented by the x-coordinate, but now the y-coordinate gives the corresponding difference in phased maternal DNM counts. As expected, no effect of paternal age on maternal DNM count is apparent in this plot. Finally, panel (d) shows the effect of maternal age on paternal DNMs. In the analysis of Gao et al.[19], this showed a weakly significant positive correlation, but in our larger dataset there is no such signal - in fact there is an apparent negative correlation, caused by the sparsity of data points at large maternal age differences.

Thus these findings are consistent with a limited impact of parental age on the number of early postzygotic mutations in the embryo.

## Supplementary Note 7. Modelling mean-variance overdispersion for DNM count data

Given that the phenotype of interest (DNM counts) in our study represents count data, a natural assumption would be that it follows a discrete distribution within the Poisson family. Overdispersion can be modelled by different distributions such as Poisson, Quasi-poisson, and negative binomial, as shown in **Supplementary Figure 20**. The quasi-Poisson distribution (line b) assumes the variance is a linear function of the mean, same as the Poisson distribution (line a), but with a slope greater than 1, and the negative binomial distribution posits a quadratic relationship between the mean and variance (line c).

To elucidate the form of overdispersion present in the phased DNM count data, we constructed a diagnostic plot of the empirical fit of the variance-mean relationship, grouping samples by parental age at conception (the major factor determining DNM rate) into twenty bins of equal age intervals. We calculated the mean of samples falling into each bin and placed them on the x-axis, while the sample variances were computed and placed on the y-axis. This plot can be observed in **Supplementary Figure 21** (A,B). Here, it can be noted that the data exhibits a linear relationship with a steeper slope than the variance = mean line. This linear trend suggests that the quasi-Poisson distribution is a more suitable model for our data than the negative binomial or Poisson distributions.

Additionally, to fit the model to the data, both quasi-Poisson and negative binomial models employ the iteratively weighted least-squares algorithm. The concept of weight here was used to take into account the unequal variance among residuals by modelling the objective function in a form of weighted least squares problem. Thus, another distinguishing factor between the quasi-Poisson and negative binomial regression models is the difference in the weights between these models. For instance, in the quasi-Poisson model, the weights are directly proportional to the mean, whereas in the negative binomial model, the weights exhibit a concave relationship with the mean, as shown in the equation below.

$$W_{Quasi-Poisson} = diag(\frac{\mu_1}{\theta} \dots \frac{\mu_n}{\theta}),$$

$$W_{Negative-binomial} = diag(\frac{\mu_1}{1+\kappa\mu_1} \dots \frac{\mu_n}{1+\kappa\mu_n}),$$

We performed both quasi-Poisson and negative binomial regression analyses on the data sets for paternally and maternally phased DNMs, respectively. In each model, we plotted the estimated weights against the fitted values. As seen in **Supplementary Figure 21** (C,D), the quasi-Poisson regression results exhibit a linear relationship, characteristic of the quasi-Poisson model, while in the case of the negative binomial model, a slight curvature is observed. Given the patterns observed in the relationship between variance and mean, as well as the changing patterns of weights, we conclude that the quasi-Poisson model is more suitable for our data [20].

## Supplementary References


1. Yoshida, K. *et al.* Tobacco smoking and somatic mutations in human bronchial epithelium. *Nature* **578**, 266–272 (2020).
2. Sondka, Z. *et al.* COSMIC: a curated database of somatic variants and clinical data for cancer. *Nucleic Acids Res.* **52**, D1210–D1217 (2024).
3. Samuels, D. C. *et al.* Heterozygosity ratio, a robust global genomic measure of autozygosity and its association with height and disease risk. *Genetics* **204**, 893–904 (2016).
4. Acuna-Hidalgo, R. *et al.* Post-zygotic point mutations are an underrecognized source of DE Novo genomic variation. *Am. J. Hum. Genet.* **97**, 67–74 (2015).
5. Wang, R. J., Al-Saffar, S. I., Rogers, J. & Hahn, M. W. Human generation times across the past 250,000 years. *Sci. Adv.* **9**, eabm7047 (2023).
6. Lesthaeghe, R. J. The second demographic transition: also a 21st century Asian challenge? *China Popul. Dev. Stud.* **6**, 228–236 (2022).
7. Harris, K. & Pritchard, J. K. Rapid evolution of the human mutation spectrum. *Elife* **6**, (2017).
8. Cooper, D. N. & Krawczak, M. Cytosine methylation and the fate of CpG dinucleotides in vertebrate genomes. *Hum. Genet.* **83**, 181–188 (1989).
9. Jónsson, H. *et al.* Parental influence on human germline de novo mutations in 1,548 trios from Iceland. *Nature* **549**, 519–522 (2017).
10. Zhou, H., He, K., Chen, J. & Zhang, X. LinDA: linear models for differential abundance analysis of microbiome compositional data. *Genome Biol.* **23**, 95 (2022).
11. Koh, G., Degasperi, A., Zou, X., Momen, S. & Nik-Zainal, S. Mutational signatures: emerging concepts, caveats and clinical applications. *Nat. Rev. Cancer* **21**, 619–637 (2021).
12. Alexandrov, L. B. *et al.* Signatures of mutational processes in human cancer. *Nature* **500**, 415–421 (2013).

13. Islam, S. M. A. *et al.* Uncovering novel mutational signatures by de novo extraction with SigProfilerExtractor. *Cell Genom* **2**, None (2022).

14. Moore, L. *et al.* The mutational landscape of human somatic and germline cells. *Nature* **597**, 381–386 (2021).

15. Li, Y. *et al.* Patterns of somatic structural variation in human cancer genomes. *Nature* **578**, 112–121 (2020).

16. Teh, Y. W., Jordan, M. I., Beal, M. J. & Blei, D. M. Hierarchical Dirichlet Processes. *J. Am. Stat. Assoc.* **101**, 1566–1581 (2006).

17. Wild, F. An LSA Package for R. (2007).

18. Mingard, C. *et al.* Dissection of Cancer Mutational Signatures with Individual Components of Cigarette Smoking. *Chem. Res. Toxicol.* **36**, 714–723 (2023).

19. Gao, Z. *et al.* Overlooked roles of DNA damage and maternal age in generating human germline mutations. *Proc. Natl. Acad. Sci. U. S. A.* **116**, 9491–9500 (2019).

20. Ver Hoef, J. M. & Boveng, P. L. Quasi-Poisson vs. negative binomial regression: how should we model overdispersed count data? *Ecology* **88**, 2766–2772 (2007).
