# Supplementary material for: The impact of ancestral, genetic, and environmental influences on germline de novo mutation rates and spectra
Source: Nat Commun. 2025 May 15;16:4527. doi: 10.1038/s41467-025-59750-x (PMC12081642; doi:10.1038/s41467-025-59750-x)
Supplement: Supplementary file 2 — Description Of Additional Supplementary Files [file 41467_2025_59750_MOESM2_ESM.pdf]

Description of additional supplementary files

Supplementary Data 1. Ancestry-associated differences in DNM counts per trio. Summary statistics for DNM count comparisons across five super-continental ancestry classifications (AFR, AMR, EAS, EUR, SAS) using a generalised linear regression (Model 1, Methods). A total of 10 comparisons were performed and obtained p values were adjusted to account for multiple testing using the Benjamini-Hochberg method (FDR). Column key: “ComparedAncestries”: ancestry pair compared; “FoldChange”: DNM count fold change difference estimate from generalised linear regression model (ancestry A / ancestry B); “log(FoldChange)”: logarithm of fold change estimate; “StandardError(logFC)”: standard error for the log fold change; “p value”: nominal two-sided p value for the effect estimate; “adjusted p”: “Benjamini–Hochberg” (FDR) adjusted p value; “significance label”: NA indicates no significant differences after p value adjustment, otherwise significance level for adjusted p is indicated.

Supplementary Data 2. Ancestry-associated differences in DNM spectra (pyrimidine substitution counts/total trio DNMs). Summary statistics for DNM spectra comparisons across five super-continental ancestry classifications (AFR, AMR, EAS, EUR, SAS) and seven pyrimidine substitution proportions using a compositional regression model (Model 2, Methods). A total of 70 comparisons were performed and obtained p values were adjusted to account for multiple testing using the Benjamini-Hochberg method (FDR). Column key: “ComparedAncestries”: ancestry pair compared; “PyrimidineSubstitution”: Tested pyrimidine substitution; “FoldChange”: Pyrimidine substitution proportion fold change difference estimate from the compositional regression model (ancestry A / ancestry B); “log2(FoldChange)”: logarithm (base 2) of fold change estimate; “StandardError(log2FC)”: standard error for the log2 fold change; “p value”: nominal two-sided p value for the effect estimate; “adjusted p”: “Benjamini–Hochberg” (FDR) adjusted p value; “significance label”: NA indicates no significant differences after p value adjustment, otherwise significance level for adjusted p is indicated.

Supplementary Data 3. Ancestry associated differences in SNP-based pyrimidine substitution spectra (derived from Harris and Pritchard, 2017 [14]). Summary statistics from chi-squared tests for mutation spectra differences across five super-continental ancestry classifications (AFR, AMR, EAS, EUR, SAS) based on population single nucleotide polymorphism (SNP) data from Harris and Pritchard, 2017 [14]**.** Column key: “reference ancestry”: ancestry used as baseline for given comparison; “compared ancestry”: ancestry compared against baseline; “pyr subs”: pyrimidine substitution proportion being compared; “fold change”: pyrimidine proportion fold change differences; “pval”: p values from two-sided chi-squared test comparison of pyrimidine substitution counts between two ancestries; “ordered p”: chi-squared test p values calculated for pyrimidine substitutions arranged by raw significance; “significance label”: comparisons with ordered p value <= 1e-4; “pyr code”: number of pyrimidine substitutions used to collapse the original 96 substitution code annotation. This can be either a 7-code (C>A, C>G, C>T, CpG>TpG, T>A, T>G, and T>C; note that C>T includes CpG>TpG) or a 6-code excluding the CpG>TpG class (i.e. not making distinction of C>T substitutions occurring in CpG sites vs those occurring elsewhere).

Supplementary Data 4. Summary of sources and number of SNPs used as instrumental variables (IVs) in Mendelian randomisation analysis. Column key: “Phenotype”: Exposure phenotype; “Study Origin”: Publication of origin of exposure IVs. “Sex-combined”: number of IVs extracted from the sex-combined DNM rate GWAS; “Father”: number of IVs extracted from the paternal DNM rate GWAS; “Mother”: number of IVs extracted from the maternal DNM rate GWAS.

Supplementary Data 5. Mendelian randomisation on paternal DNM rate. Summary statistics of Mendelian randomisation testing for the causal effect of exposures (namely: Age of initiation of smoking, Genital disorder, Hydrocele spermatocele, Age at natural menopause, Sleep duration, Smoking cessation, Smoking initiation, BMI, Drinks per week) on paternal DNM rate. Estimates were obtained using four methods (namely: Simple median, Weighted median, IVW, MR-Egger). MR effect estimate p-values are two-tailed and not adjusted for multi-testing correction. Column key: **“**Method”: Method used for Mendelian Randomisation. “Std.Error”: Standard error for MR effect estimate. “95% CI lower”: 95% confidence interval for MR effect estimate. “95% CI upper“: 95% confidence interval for MR effect estimate. “p value”: MR effect estimates nominal p value. “Exposure”: Tested phenotype exposure. “LD”: Linkage disequilibrium threshold for instrumental variable pruning.

Supplementary Data 6. Mendelian randomisation on maternal DNM rate. Summary statistics of Mendelian randomisation testing for the causal effect of exposures (same as Supplementary Data 5) on maternal DNM rate. Column contents match those of Supplementary Data 5.

Supplementary Data 7. Mendelian randomisation on combined DNM rate. Summary statistics of Mendelian randomisation testing for the causal effect of exposures (same as Supplementary Data 5) on DNM rate across all individuals. Column contents match those of Supplementary Data 5.

Supplementary Data 8. **Association between ancestry-stratified DNM counts per trio and the number of heterozygous sites (n HETs) per parent.** Generalised linear regression p-values for the effect of parental n HETs (Supplementary Model 1, Supplementary Note 1C) on DNM counts. Column key: “Ancestry”: Tested ancestry group; “mean parental nHETs”: Association p value between number of DNMs per trio and the mean N HETs per parent; “maternal nHETs”: Association p value between number of DNMs per trio and maternal N HETs; “paternal nHETs”: Association p value between number of DNMs per trio and paternal N HETs.

Supplementary Data 9. Smoking-associated differences in DNM spectra (pyrimidine substitution counts / total phased DNMs per parent). Summary statistics for DNM spectra differences between smokers and non-smokers across seven pyrimidine substitution proportions using the compositional regression model described in Supplementary Note 4 (Supplementary Model 2). Obtained p values were adjusted to account for multiple testing using the Benjamini-Hochberg method (FDR). Column key: “PyrimidineSubstitution”: Tested pyrimidine substitution; “FoldChange”: Pyrimidine substitution proportion fold change difference estimate from the compositional regression model (smoker / non-smoker); “log2(FoldChange)”: logarithm (base 2) of fold change estimate; “StandardError(log2FC)”:

standard error for the log2 fold change; “p value”: nominal two-sided p value for the effect estimate; “adjusted p”: “Benjamini–Hochberg” (FDR) adjusted p value; “significance label”: NA indicates no significant differences after p value adjustment.

Supplementary Data 10. Summary of number of mutations and samples used for signature extraction and deconvolution to detect potential mutational signatures associated with smoking. Column key: “Origin”: Sample origin, either GEL (pooled individuals in this study), or Yoshida et al. 2020 for our external reference sample set; “donor”: Donor name for each of the sample sets shown in Supplementary Figure 4 (X-axis identifier); “smoker status”: smoker status of the sample in question, for samples taken from Yoshida et al. 2020[51] samples, “non-smoker” corresponds to the “never smoker” tag in the original publication, while “smoker” corresponds to the “current smoker” tag, respectively; “n inds pooled”: total individuals pooled for each of the GEL meta-samples; “n samples” number of samples included for each donor (bronchial epithelium samples in the case of Yoshida et al., 2020 samples), set to NA for GEL meta-individuals; “n mutations”: Total number of mutations included for each donor, in the case of GEL samples, this corresponds to the number of pooled DNMs per meta-individual.

Supplementary Data 11. Summary statistics for sex-combined DNM rate GWAS. DNM counts per individual (i.e., phased DNMs) were residualised according to Model 3. Residualised DNM counts were used as phenotypes for GWAS across 15,885 individuals genetically identified as European descendants (Methods). Column key: “chrom”: chromosome, “pos”: GRCh38 assembly position. “marker.id”: Construct of “chrom:pos_other.allele_effect.allele”. “other.allele”: allele NOT used for regression estimate. “effect.allele”: Allele used as reference for beta effect; “af_effect.allele”: Sample subset specific allele frequency of the effect allele. “Beta”: Regression beta effect (SAIGE); “se”: Standard error for beta effect; “p.value” P value for beta effect. This data is available in the GWAS catalogue (https://www.ebi.ac.uk/gwas/home) under the accession number GCST90565198, and in figshare under the DOI https://doi.org/10.6084/m9.figshare.28633352.

Supplementary Data 12. Summary statistics for paternal DNM rate GWAS. Paternally phased DNM counts were residualised according to Model 4 (Methods). Residualised DNM counts were used as phenotypes for GWAS across 7,982 individuals (trio fathers genetically identified as European descendants, Methods). Column contents match those of Supplementary Data 10. Column contents match those of Supplementary Data 10. This data is available in the GWAS catalogue (https://www.ebi.ac.uk/gwas/home) under the accession number GCST90565197, and in figshare under the DOI https://doi.org/10.6084/m9.figshare.28633352.

Supplementary Data 13. Summary statistics for maternal DNM rate GWAS. Maternally phased DNM counts were residualised according to Model 5 (Methods). Residualised DNM counts were used as phenotypes for GWAS across 7,993 individuals (trio mothers genetically identified as European descendants, Methods). Column contents match those of Supplementary Data 10. Column contents match those of Supplementary Data 10. This data is available in the GWAS catalogue (https://www.ebi.ac.uk/gwas/home) under the accession number GCST90565196, and in figshare under the DOI https://doi.org/10.6084/m9.figshare.28633352.
