# Supplementary material for: The impact of ancestral, genetic, and environmental influences on germline de novo mutation rates and spectra
Source: Nat Commun. 2025 May 15;16:4527. doi: 10.1038/s41467-025-59750-x (PMC12081642; doi:10.1038/s41467-025-59750-x)
Supplement: Supplementary file 4 — Reporting Summary [file 41467_2025_59750_MOESM4_ESM.pdf]

nature portfolio

Corresponding author(s): Hilary C. Martin
Raheleh Rahbari
Aylwyn Scally

Last updated by author(s): 15/04/25

Reporting Summary

Nature Portfolio wishes to improve the reproducibility of the work that we publish. This form provides structure for consistency and transparency in reporting. For further information on Nature Portfolio policies, see our Editorial Policies and the Editorial Policy Checklist.

Statistics

For all statistical analyses, confirm that the following items are present in the figure legend, table legend, main text, or Methods section.

n/a	Confirmed	
☐	☒	The exact sample size (*n*) for each experimental group/condition, given as a discrete number and unit of measurement
☐	☒	A statement on whether measurements were taken from distinct samples or whether the same sample was measured repeatedly
☐	☒	The statistical test(s) used AND whether they are one- or two-sided<br>*Only common tests should be described solely by name; describe more complex techniques in the Methods section.*
☐	☒	A description of all covariates tested
☐	☒	A description of any assumptions or corrections, such as tests of normality and adjustment for multiple comparisons
☐	☒	A full description of the statistical parameters including central tendency (e.g. means) or other basic estimates (e.g. regression coefficient) AND variation (e.g. standard deviation) or associated estimates of uncertainty (e.g. confidence intervals)
☐	☒	For null hypothesis testing, the test statistic (e.g. *F*, *t*, *r*) with confidence intervals, effect sizes, degrees of freedom and *P* value noted<br>*Give P values as exact values whenever suitable.*
☒	☐	For Bayesian analysis, information on the choice of priors and Markov chain Monte Carlo settings
☒	☐	For hierarchical and complex designs, identification of the appropriate level for tests and full reporting of outcomes
☐	☒	Estimates of effect sizes (e.g. Cohen's *d*, Pearson's *r*), indicating how they were calculated

Our web collection on statistics for biologists contains articles on many of the points above.

Software and code

Policy information about availability of computer code

Data collection	NA
Data analysis	Plink (v.1.9) was used to process genotype data. Samtools (v.1.15.1) and jvarkit (https://github.com/lindenb/jvarkit) were used to extract read level information. SNP heritability was estimated using GCTA (v1.94.0). SAIGE (v.1.07) was used to perform GWAS. Instrumental variable LD-pruning was performed using LDlinkR (v1.2.2). Two sample Mendelian Randomization was performed using the MendelianRandomization (v.0.9.0) R package. De novo mutational signature annotation was performed using the “hdp” R package (https://github.com/nicolaroberts/hdp). Mutational signature deconvolution was performed using SigProfilerExtractor. All remaining analyses were performed in R (v.4.2.1). We have deposited a copy of the code used to generate our regression analyses in github (https://github.com/isaacg322/EnvGenDNM). This code is also available and fully reproducible within the Genomics England research environment (/re_gecip/shared_allGeCIPs/aeg_dnm_2024).

For manuscripts utilizing custom algorithms or software that are central to the research but not yet described in published literature, software must be made available to editors and reviewers. We strongly encourage code deposition in a community repository (e.g. GitHub). See the Nature Portfolio guidelines for submitting code & software for further information.

Data

Policy information about availability of data

All manuscripts must include a data availability statement. This statement should provide the following information, where applicable:

- Accession codes, unique identifiers, or web links for publicly available datasets
- A description of any restrictions on data availability
- For clinical datasets or third party data, please ensure that the statement adheres to our policy

Whole-genome sequence data and phenotypic data from the 100,000 Genomes project can be accessed by application to Genomics England (https://www.genomicsengland.cgfbo.uk/research/academic/join-gecip). GWAS summary statistics of DNM rate generated in this study are available in figshare under the DOI https://doi.org/10.6084/m9.figshare.28633352 and the GWAS catalogue (https://www.ebi.ac.uk/gwas/home) under the accession numbers: GCST90565198 (sex combined DNM rate), GCST90565197 (paternal DNM rate), and GCST90565196 (maternal DNM rate). Publicly available GWAS summary statistics can be accessed at various resources: http://geneatlas.roslin.ed.ac.uk, https://conservancy.umn.edu/handle/11299/241912, https://www.reprogen.org/. Somatic mutations from ascertained smoker individuals can be accessed at:https://data.mendeley.com/datasets/b53h2kwpyy/2. Reference single base substitution mutational signatures used for deconvolution can accessed at: https://cancer.sanger.ac.uk/signatures/sbs/

Research involving human participants, their data, or biological material

Policy information about studies with human participants or human data. See also policy information about sex, gender (identity/presentation), and sexual orientation and race, ethnicity and racism.

Reporting on sex and gender	We used biological sex reported by 100,000 genomes project participants metadata. Most of the analyses were performed in both sex combined, and sex specific subsets (smoking regressions, heritability estimation, GWAS, and Mendelian Randomisation). Sample sizes can be found in the Methods section.
Reporting on race, ethnicity, or other socially relevant groupings	Ancestry classifications were available as part of the 100,000 genomes project metadata. Here, ancestry was defined by genetic similarity to individuals in the 1000 Genomes project, in turn classified in one of five continental-level populations. For GWAS, heritabilty estimation, MR, and de novo mutational signature annotation we restricted to individuals with EUR genetic ancestry. Sample sizes can be found in the Methods section. We used 20 genetic principal components to adjust for fine-scale population structure in GWAS.
Population characteristics	Individuals were recruited as part of the rare disease programme from the Genomics England initiative across 9 British hospitals. Recruited individuals presented a “rare disease”, which is defined as a disorder affecting <= 1 person in 2000 people
Recruitment	The 100,000 Genomes project recruited rare disease families and cancer patients through NHS. The UKHLS cohort aimed to capture a representative sample of people living in the UK and to collect longitudinal socioeconomic and other data on them
Ethics oversight	The 100,000 Genomes project was approved by the East of England—Cambridge Central Research Ethics Committee (REF 20/EE/0035).

Note that full information on the approval of the study protocol must also be provided in the manuscript.

Field-specific reporting

Please select the one below that is the best fit for your research. If you are not sure, read the appropriate sections before making your selection.

☒ Life sciences ☐ Behavioural & social sciences ☐ Ecological, evolutionary & environmental sciences

For a reference copy of the document with all sections, see nature.com/documents/nr-reporting-summary-flat.pdf

Life sciences study design

All studies must disclose on these points even when the disclosure is negative.

Sample size	Sample sizes were determined by the maximum number of individuals with complete sequencing statistics, de novo variant calling metadata, and (where relevant) de novo mutation phasing information.
Data exclusions	We excluded siblings in multiplex families. We kept only the de novo mutations detected in the offspring conceived at the latest parental age in such cases.
Replication	Replication was not possible as a cohort of similar size and diversity is not yet publicly available
Randomization	No randomisation of participants was performed in this study.
Blinding	Blinding was not possible because analysts needed to use the phenotype data in the analysis, or perform analysis in a subset of the participants with a certain characteristic (e.g participants with smoking records).

Reporting for specific materials, systems and methods

We require information from authors about some types of materials, experimental systems and methods used in many studies. Here, indicate whether each material, system or method listed is relevant to your study. If you are not sure if a list item applies to your research, read the appropriate section before selecting a response.

Materials & experimental systems

n/a	Involved in the study
☒	☐ Antibodies
☒	☐ Eukaryotic cell lines
☒	☐ Palaeontology and archaeology
☒	☐ Animals and other organisms
☒	☐ Clinical data
☒	☐ Dual use research of concern
☒	☐ Plants

Methods

n/a	Involved in the study
☒	☐ ChIP-seq
☒	☐ Flow cytometry
☒	☐ MRI-based neuroimaging

Plants

Seed stocks	*Report on the source of all seed stocks or other plant material used. If applicable, state the seed stock centre and catalogue number. If plant specimens were collected from the field, describe the collection location, date and sampling procedures.*
Novel plant genotypes	*Describe the methods by which all novel plant genotypes were produced. This includes those generated by transgenic approaches, gene editing, chemical/radiation-based mutagenesis and hybridization. For transgenic lines, describe the transformation method, the number of independent lines analyzed and the generation upon which experiments were performed. For gene-edited lines, describe the editor used, the endogenous sequence targeted for editing, the targeting guide RNA sequence (if applicable) and how the editor was applied.*
Authentication	*Describe any authentication procedures for each seed stock used or novel genotype generated. Describe any experiments used to assess the effect of a mutation and, where applicable, how potential secondary effects (e.g. second site T-DNA insertions, mosiacism, off-target gene editing) were examined.*
